# Rapid Decentralized Prostate Cancer Risk Stratification by Portable Liquid Biopsy Analysis within a Clinical Biosensor Validation Framework

**DOI:** 10.1002/advs.202512126

**Published:** 2026-01-27

**Authors:** Kevin M. Koo, Grant Phillips, Sriganesh Srihari, Áine Farrell, Binny Jaradi, Kira J. Fitzpatrick, John W. Yaxley, Hemamali Samaratunga, Paul N. Mainwaring, Ke‐lin Ru, Darren J. Korbie, Scott A. Tomlins, Matthew J. Roberts, Robert A. Gardiner, Matt Trau

**Affiliations:** ^1^ The University of Queensland Centre for Clinical Research (UQCCR) Brisbane QLD Australia; ^2^ XING Applied Research & Assay Development (XARAD) Division XING Technologies Pty Ltd Brisbane QLD Australia; ^3^ Centre for Personalized Nanomedicine Australian Institute for Bioengineering and Nanotechnology (AIBN) The University of Queensland Brisbane QLD Australia; ^4^ QIMR Berghofer Centre for Immunotherapy and Vaccine Development and Translational and Human Immunology Laboratory Department of Immunology QIMR Berghofer Medical Research Institute Brisbane QLD Australia; ^5^ Department of Urology Royal Brisbane and Women's Hospital Brisbane QLD Australia; ^6^ Aquesta Specialized Uropathology Brisbane QLD Australia; ^7^ Faculty of Health, Medicine and Behavioural Sciences The University of Queensland Brisbane QLD Australia; ^8^ Department of Pathology University of Michigan Medical School Ann Arbor MI USA; ^9^ Strata Oncology Ann Arbor MI USA; ^10^ Edith Cowan University Perth WA Australia; ^11^ School of Chemistry and Molecular Biosciences The University of Queensland Brisbane QLD Australia

**Keywords:** prostate cancer, liquid biopsy, clinical validation, biomarkers, biosensor

## Abstract

Clinical liquid biopsy (e.g., blood or urine) specimens, as a minimally invasive source of crucial molecular information, continue to grow in importance for efficacious targeted cancer treatment. Yet, current molecular profiling technologies are still confined to centralized laboratory testing, which escalates testing costs, result turnaround time, and patient anxiety. Crucially, there is also a scarcity of purposeful clinical validation studies to rigorously evaluate emerging liquid biopsy technologies from research settings to facilitate clinical translation. Here, we report liquid biopsy biosensor advancements in achieving rapid, accurate, and decentralized molecular profiling of a clinically accredited prostate cancer (PCa) urinary circulating RNA biomarker panel. This biosensor approach, termed ‘Accelerated non‐inVasive bioAnalyte testing And Reporting’ (AVATAR), integrates accelerated isothermal assay chemistry and wireless mobile operation capabilities onto a portable electrochemical readout platform. AVATAR enables easy operational control and result display with a custom mobile app following liquid biopsy specimen collection. Using independent training (*n* = 124) and validation (*n* = 114) PCa clinical urinary specimen cohorts, we showed AVATAR achieved superior PCa risk stratification to current clinical PCa testing with area‐under‐curve values of 0.88 (95% confidence interval: 0.84−0.92) and 0.86 (95% confidence interval: 0.83−0.89), respectively within 55 min of assay time. To further accelerate AVATAR for using PCa liquid biopsies as a surrogate for invasive tissue sampling, we designed a tailored multi‐year PCa follow‐up study with individual patient liquid biopsy specimens showing strong molecular biomarker correlation with comprehensive transcriptomic sequencing of matched tissue specimens (*n* = 39). By creating this bespoke biosensor technology clinical translation framework, we demonstrated AVATAR for decentralized PCa liquid biopsy molecular profiling to augment clinical precision cancer management planning.

## Graphical abstract

In this work, we showcased two significant scientific advances in (i) developing a portable biosensor technology for rapid decentralized prostate cancer urinary biomarker testing and reporting, with superior performance to current clinical testing practice; and (ii) proposing a clinical validation framework (for our biosensor development) which can be widely utilized to promote clinical translation of biosensor technologies in academia.

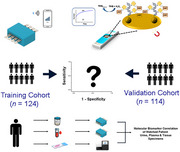

## 1 Introduction

Liquid biopsies are becoming an increasingly valuable class of minimally‐invasive circulating cancer biomarkers for both cancer diagnosis and prognosis [1, 2, 3, 4, 5]. Traditional tissue biopsies are generally invasive surgical procedures with possible side effects, whereas liquid biopsies are readily accessible in the form of blood or urine for widespread repeated use, particularly benefitting cancer patients who may be ineligible for tissue biopsy procedures. Furthermore, as true for many solid cancers, tumor heterogeneity and needle biopsy sampling limitations mean that a true molecular snapshot is not possible; whereas liquid biopsies comprise overall molecular information from different cancer cells to enable effective targeted cancer treatment through molecular profiling [6]. Of note, liquid biopsies can help delineate prostate cancer (PCa), the paradigm cancer exemplar for intra‐ and inter‐tumoral molecular heterogeneity [7, 8, 9, 10, 11, 12], for better clinical management outcomes.

PCa is one of the most common and deadly malignancies in men worldwide [13, 14]. For several decades now, the clinical diagnosis and prognosis of PCa have been dependent on histopathological assessment of biopsied tissue samples after initial detection of an elevated prostate‐specific antigen (PSA) level in blood. However, this practice remains increasingly controversial due to the consensus that PSA testing has led to excessive PCa overdiagnosis and overtreatment, as well as an inability to accurately risk‐stratify high‐ and low‐risk disease molecular subtypes [15]. This clinical challenge is mainly due to the limitations in PSA as a PCa biomarker [16, 17], in both clinical specificity (elevated PSA levels due to non‐cancerous conditions) and sensitivity (significant PCa present at “normal” PSA levels); resulting in men being subjected to unnecessary treatments which include risks of urinary incontinence, proctitis, sexual dysfunction, infection and suicide. Additionally, the typical PSA biomarker testing and reporting workflow generally involves a patient blood sample to be collected and sent to a centralized laboratory for analysis before the test result is reported to the clinician for decision‐making. The entire process requires various personnel with ensuing financial burden and a result turnaround delay time that can cause anxiety for patients. Thus, innovative decentralized accurate testing with PCa‐specific biomarkers in minimally invasive patient liquid biopsies is required as an accessible point‐of‐need approach to improve the status‐quo of initial (and subsequent) clinical PCa management [18].

To address the challenges of achieving improved minimally invasive PCa testing and reporting using patient liquid biopsies, we envisage that a biosensor approach can combine rapid biomarker sensing chemistry with a compact miniaturized readout and instantaneous result reporting [19, 20, 21, 22, 23]. Although the concept of nanobiosensing is well introduced, such a technology is yet to be deployed for point‐of‐need clinical testing of PCa liquid biopsies. Herein, we report on a miniaturized bio‐detector/‐reporter device—termed ‘Accelerated non‐inVasive bioAnalyte Testing And Reporting’ (AVATAR)–for rapid point‐of‐need profiling of multiple PCa‐specific RNA biomarkers in liquid biopsy samples. Specifically, AVATAR leverages rapid assay chemistry and mobile digital technology to detect and wirelessly report a panel of PCa liquid biopsy biomarker signature on a miniaturized device platform.

Despite the incentive to push for the use of liquid biopsies in PCa management, there is a general lack of purposeful clinical validation studies to rigorously evaluate the combination of liquid biopsy biomarkers and related detection technologies [24]. Importantly, this leads to a gap between biosensor research and translation into real‐world use/impact, thus slowing the translation of applied knowledge into effective new health measures. In comparison to existing biosensor technologies, this study exhibits distinct innovations in circulating RNA biomarker profiling and biodevice engineering, focusing on robust biosensor technology validation studies for clinical use translation. In this work, we explored a multidisciplinary research pipeline: i) design and development of AVATAR platform technology to address the unmet need of rapid decentralized PCa liquid biopsy biomarker testing with excellent single cell‐level analytical detection sensitivity, specificity and repeatability; ii) Testing of a panel of six clinically accredited urinary RNA biomarkers on AVATAR for accurate PCa diagnosis and disease risk stratification in independent training (*n* = 124) and validation cohorts (*n* = 114); iii) Correlation of molecular biomarker profiling in matched (*n* = 39) liquid biopsy (plasma and urine) and tissue specimens from individual PCa patients in a tailored multi‐year PCa follow‐up study in order to robustly evaluate biosensor‐driven PCa liquid biopsy as a viable clinical alternative to tissue biopsy biomarker profiling. With the biosensor technology and clinical advances showcased through devising AVATAR for improved clinical liquid biopsy biomarker profiling, this body of work has the potential to accelerate the transformation of PCa clinical management.

## 2 Results

### 2.1 AVATAR Technology Development

Our AVATAR technology is made up of a miniaturized detector/reporter device with wireless mobile connectivity, and reciprocal assay chemistry for rapid isothermal recombinase polymerase amplification (RPA) and chronoamperometric measurement of different PCa urinary biomarkers. To promote clinical utility, we elected to test for a panel of six urinary PCa RNA biomarkers, which have been clinically validated in multiple international patient cohorts and are currently clinically accredited for non‐invasive, clinically significant PCa testing–MyProstate Score (Lynx Dx) and SelectMdx (MDxHealth) [25, 26, 27, 28, 29, 30, 31]. These urinary biomarkers include *TMPRSS2‐ERG* (T2:ERG), *PCA3*, *SChLAP1*, *HOXC6*, *DLX1*, *KLK2*.

**FIGURE 1:**
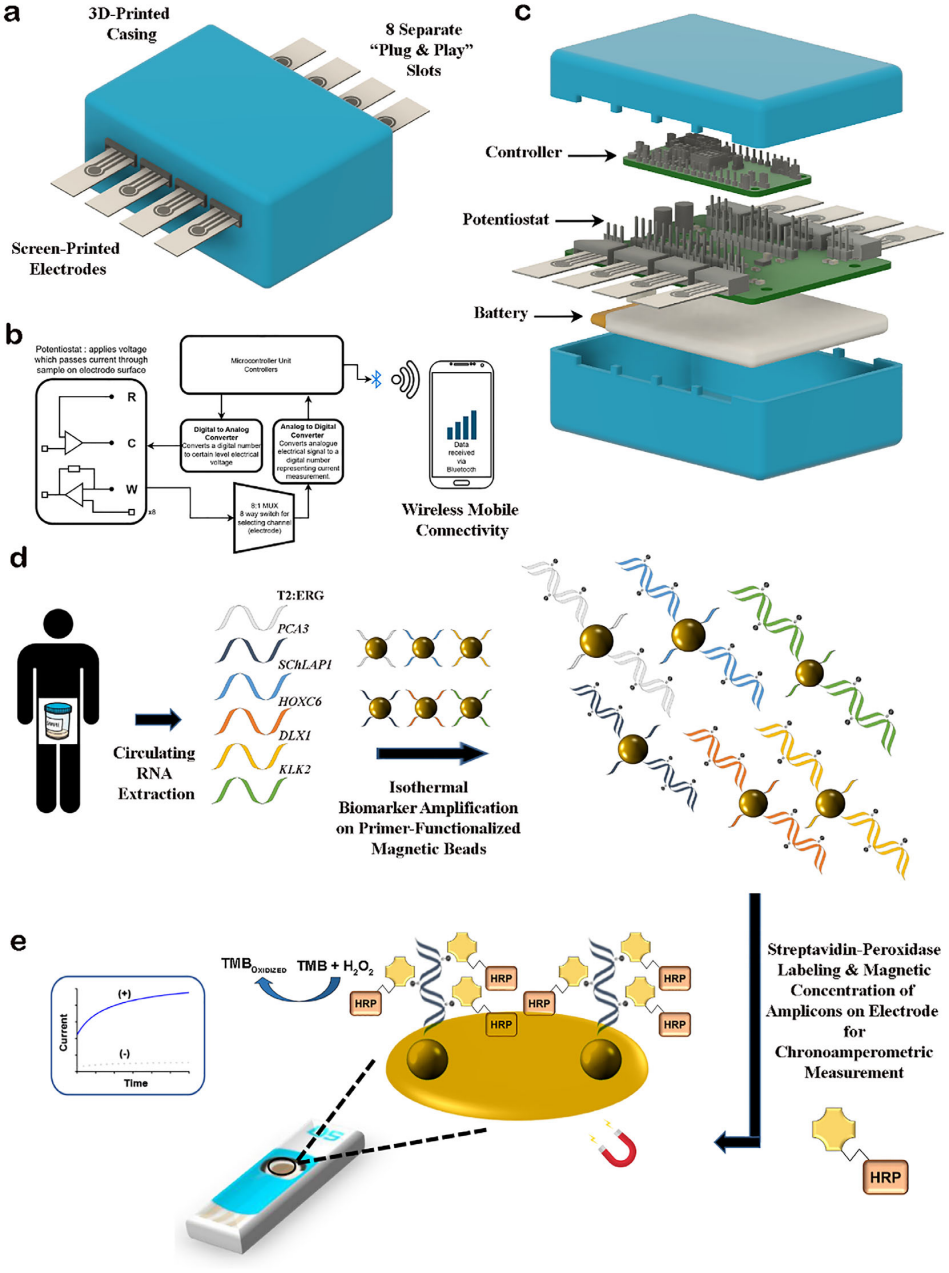
AVATAR (Accelerated noninvasive bioanalyte Testing and Reporting), a miniaturized biosensor device for rapid decentralized profiling of clinically accredited RNA biomarkers in patient urine/plasma specimens. (a) The AVATAR device is palm‐sized with eight separate slots for individual electrode insertion and output signal measurement. (b) Each slot is linked to a potentiostat capable of measuring a wide range of current (±7.5 µA). The eight potentiostats were connected to a digital‐to‐analog converter for potential control, an analog‐to‐digital converter for signal digitization, a multiplexer for channel selection, and a microcontroller unit for system operation. An external smartphone with a customized app can communicate with the AVATAR device wirelessly via Bluetooth for operational control and data logging. (c) The AVATAR device is designed to be encased within a 3D‐printed casing for use with up to eight “plug‐and‐play” screen‐printed electrodes simultaneously. This design offers high scalability and flexibility of various array size formats with rapid chronoamperometric readout. (d) Circulating RNA is first extracted from patient liquid biopsy samples. Primer‐functionalized magnetic beads specific for each of the six biomarkers (T2:ERG, *PCA3*, *SChLAP1*, *HOXC6*, *DLX1*, *KLK2*) are introduced to initiate isothermal amplification of biomarkers on the bead surfaces. Biotin‐modified uracil bases are randomly incorporated into the amplicons. (e) The magnetic bead‐bound amplicons are labeled with peroxidase enzymes via biotin‐streptavidin bonds and magnetically concentrated on the electrode surface. The electrode is plugged into the AVATAR device to initiate chronoamperometric readout, and the resultant current signal (corresponding to biomarker level) is measured. HRP, horseradish peroxidase; TMB, 3,3′,5,5′‐tetramethylbenzidine; H_2_O_2,_ hydrogen peroxide.

The AVATAR device is constructed to be of palm‐sized equipment footprint (10 cm by 6 cm by 2 cm) with eight separate slots for insertion and output signal measurement of individual assays on independent gold electrode surfaces (Figure 1a). For broad applicability, the slots were designed to function with single‐use screen‐printed electrodes to avoid sensor surface biofouling due to repeat usage (Figure S1). Each slot is linked to a potentiostat capable of measuring a wide range of current (±7.5 µA). The eight potentiostats were connected to a digital‐to‐analog converter for potential control, an analog‐to‐digital converter for signal digitization, a multiplexer for channel selection, and a microcontroller unit for system operation (Figure 1b). It is noteworthy that the chronoamperometric readout of our AVATAR device (via “plug‐and‐play” screen‐printed electrodes) offers high scalability and flexibility of various array size formats (Figure 1c). Most importantly, the AVATAR device is designed to accommodate the AVATAR assay workflow on the screen‐printed electrode surface for multiple biomarkers simultaneously.

To initiate the AVATAR assay, total RNA is first extracted from various liquid biopsy samples and isothermally amplified on specific primer‐functionalized magnetic beads (Table S1). The advantages of isothermal RPA on magnetic beads are 1) rapid ease of magnetic amplicon purification post‐amplification, 2) no need for time‐consuming electrode surface functionalization for target capture, 3) better electrochemical signal reproducibility without variable electrode surface modifications. In all, six different sets of magnetic nanoparticles were pre‐prepared for parallel isothermal RPA for each of the six RNA biomarkers. During isothermal RPA, biotin‐modified uracil bases are randomly incorporated into the newly synthesized amplicon strands to serve as binding sites for attaching streptavidin‐modified peroxidase enzymes (Figure 1d) [32, 33]. Post‐isothermal RPA, the amplicons are magnetically concentrated onto the surfaces of screen‐printed electrodes for biosensing without tedious electrode surface pre‐modification and related signal variabilities. Finally, TMB (3,3′,5,5′‐tetramethylbenzidine) was added as a chromogenic electron mediator to generate a peroxidase‐catalyzed current signal, which in turn directly signifies the corresponding biomarker level (Figure 1e). Hence, this permits a miniaturized portable device alternative to conventional laboratory‐based thermocycling and electrochemical readout instruments for decentralized molecular biomarker analysis.

To facilitate decentralized point‐of‐need testing and reporting, we further developed wireless mobile‐based operation of the AVATAR device for chronoamperometric measurements [34, 35]. The detection with a smartphone is to demonstrate the feasible improvement of decentralized PCa testing for faster result turnaround time, as compared to the typical PSA biomarker testing and reporting workflow (which generally involves a patient blood sample to be collected and sent to a centralized laboratory for analysis before the test result is reported to the clinician for decision‐making). A customized app capable of communicating with the AVATAR device via Bluetooth is first designed and pre‐installed on a conventional smartphone. The compatible use of a conventional smartphone with our custom‐built AVATAR device, is to enable wireless operation of the AVATAR device for chronoamperometric measurements via a customized app. Using the app, we can activate the generation of desired voltage in individual AVATAR device slots for chronoamperometric measurements. Moreover, the resultant measurements are transmitted in real‐time from the AVATAR device to the mobile app for onboard data display; thus, omitting the prolonged result turnaround time from centralized testing facilities, and enabling prompt clinical diagnosis and any disease management action. In the age of fast information, AVATAR provides an accessible convenience for liquid biopsy molecular profiling, which is not offered by conventional biosensor technologies.

### 2.2 AVATAR Analytical Detection Performance Evaluation

We first thoroughly evaluated the analytical detection performance of AVATAR for detection specificity, sensitivity, and repeatability for the panel of six PCa biomarkers (T2:ERG, *PCA3*, *SChLAP1*, *HOXC6*, *DLX1*, *KLK2*).

**FIGURE 2:**
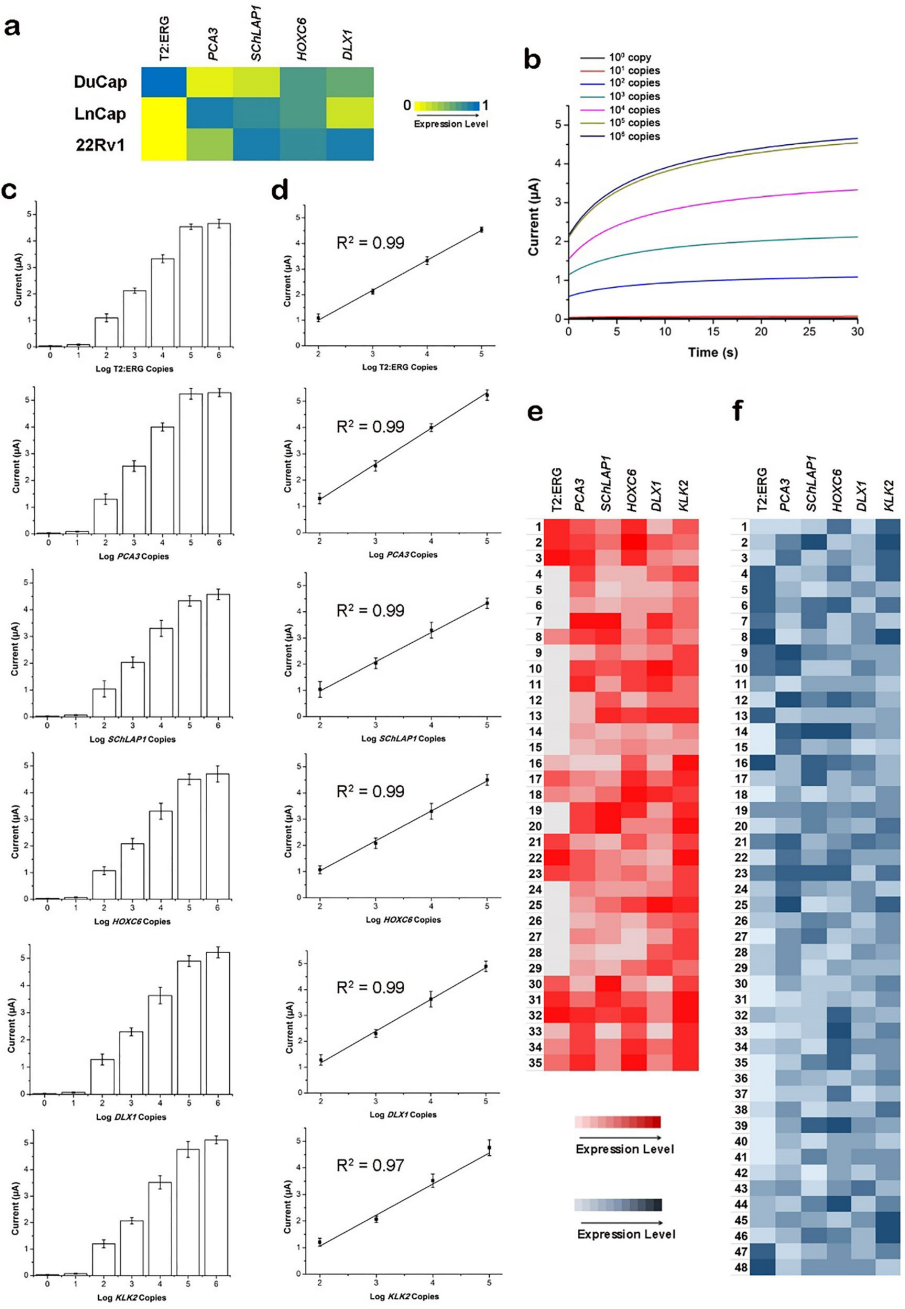
Evaluation of AVATAR analytical detection performance. (a) Detection specificity of biomarker expression levels in DuCap, LnCap, 22Rv1 prostate cancer cell lines. Six different primer sets specific to each biomarker (T2:ERG, *PCA3*, *SChLAP1*, *HOXC6*, *DLX1*, *KLK2*) were used to generate amplicons isothermally prior to chronoamperometric measurements on AVATAR. The detection specificities of all cell line biomarker expression levels are well supported by prior cell line characterization and validation data. All signal measurements were performed in triplicate and normalized against *KLK2* expression level to presented as a heat map. The data are presented as mean values. (b) Chronoamperometric signals corresponding to a serial dilution (1 to 10^6^ copies) of biomarker copies. Representative chronoamperometric signals for detection of different amounts of T2:ERG in vitro transcripts. Current is found to increase with higher biomarker copies within a serial dilution series (0 to 10^6^ copies). (c) Detection sensitivity for biomarker panel. The limit‐of‐quantification is found to be 100 copies for all six biomarkers. (d) Linear dynamic range for biomarker panel. The linear dynamic range of quantification is found to be within 10^2^–10^5^ copies for all six biomarkers. All measurements were performed in triplicate and the data are presented as mean ± s.d. in **c** and **d**. Matrix effect assessment using characterized clinical tissue and urine specimens. AVATAR biomarker analysis outcomes of patient (e) tumor tissue (*n* = 35) and (f) urine (*n* = 48) specimens are found excellent correlation (Pearson's correlation coefficient = 0.94) with targeted RNA sequencing characterization. All signal measurements were performed in triplicate and presented as heat maps. The data are presented as mean values.

The detection specificity for each of the six biomarkers was first tested by checking the isothermal amplicons for each biomarker. Utilizing stringent primer design and testing on different PCa cell lines, we confirmed that each biomarker amplification reaction has specifically generated corresponding amplicons of the expected base pair size from a heterogeneous mixture of total RNA extract from PCa cell lines (Figure S2). Next, we used three well‐characterized PCa cell lines (DuCap, LnCap, 22Rv1) as pre‐clinical biological models to further evaluate the detection specificity of our AVATAR assay. In agreement with cell line characterization data by previous studies [36, 37, 38, 39, 40], we found that normalized (to *KLK2*) biomarker expression levels showed elevated T2:ERG, *HOXC6*, and *DLX1* levels for DuCap; elevated *PCA3*, *SChLAP1*, and *HOXC6* levels for LnCap; and elevated *SChLAP1*, *HOXC6* and *DLX1* levels for 22Rv1 (Figure 2a; Figure S3). Further data validation by qPCR and gel electrophoresis of amplicons (Figure S4) also validated the biomarker expressions levels of the three cell lines. In all, we demonstrated that the specific quantification capability of our custom‐built AVATAR device with regard to isothermal RPA, labeling, and chronoamperometric readout.

The main bottleneck of qPCR‐based assays is the prolonged thermocycling process required for nucleic acid strand separation, polymerization, and fluorescence readout, whereby various temperatures must be adjusted precisely at each step. Isothermal RPA can remove the need for thermocycling, and chronoamperometric readout allows for rapid quantitative biomarker level measurements, which is ideal for affordable decentralized testing without sophisticated wavelength filters/sensors of fluorescence readout. For optimal AVATAR performance, we sequentially evaluated a range of primer concentrations (300, 325, 350, 375, 400 nm), biotin‐modified uracil base concentrations (10, 15, 20, 25, and 30 nm), magnetic bead volumes (1.2, 2.5, 5.0, 7.5, and 10.0 µL), isothermal RPA times (0, 5, 10, 15, 20, and 25 min), TMB incubation times (1, 3, 5, and 7 min) and voltages (50, 100, 150, and 200 mV). We found that a combination of 375 nm primer concentration (Figure S5), 20 nm biotin‐modified uracil base concentration (Figure S6), 5.0 µL magnetic bead volume (Figure S7), 20 min isothermal RPA time (Figure S8), 5 min TMB incubation time (Figure S9), and a constant voltage of 150 mV (Figure S10) provided the maximum current signal for biomarker detection on AVATAR.

The detection sensitivity for each of the six PCa biomarkers was assessed using serial dilutions (0 to 10^6^ copies) of T2:ERG, *PCA3*, *SChLAP1*, *HOXC6*, *DLX1*, *KLK2* in vitro transcripts (Figure 2b). It was found that AVATAR consistently achieved a limit of quantification (LoQ)—the lowest concentration within the linear range where a sample can be reliably measured with acceptable precision and accuracy—of 100 copies (Figure 2c) within a linear dynamic range of 10^2^–10^5^ copies (Figure 2d) for T2:ERG, *PCA3*, *SChLAP1*, *HOXC6*, *DLX1*, *KLK2*. It is noteworthy that the 100‐copies LoQ is adequate for circulating RNA biomarker detection at the single cell‐level [41] and thus feasible for use in clinical patient specimens. We attributed this excellent detection sensitivity of AVATAR to both molecular and readout signal enhancements by isothermal biomarker amplification on magnetic nanoparticles, and peroxidase‐catalyzed TMB oxidation (to generate electrochemically active products for chronoamperometric measurements), respectively.

As compared to gold standard qPCR, which relies on conventional PCR and fluorescence dyes for signal amplification, AVATAR has advantages in 1) rapid target amplification at a single temperature without the need for prolonged thermocycling, and 2) no need for costly fluorescence labels and sophisticated wavelength filter‐based readout instruments. Conversely, AVATAR is limited in one‐pot multiplexing capability as compared to qPCR. However, we circumvent this limitation by designing the AVATAR platform, which enables scalable, high‐throughput parallel electrochemical target detection. Our achieved detection sensitivity is equivalent to the exceptional clinically relevant detection sensitivities of recently reported isothermal electrical signaling‐based assays for circulating RNA detection in clinical cancer specimens (Table S2) [42, 43, 44, 45, 46, 47]. However, it is noteworthy that AVATAR has uniquely demonstrated molecular profiling of clinically validated PCa urinary biomarkers with a palm‐sized potentiostat in under an hour.

To investigate the detection repeatability, the coefficients of variability (%CV) over three technical/biological replicates were calculated using PCa cell lines. AVATAR displayed good assay repeatability with mean intra‐ and inter‐assay %CV of 7.7% and 8.9%, respectively. This demonstrated that the isothermal RPA and chronoamperometric readout can aid in reducing common sources of equipment error by reducing overall thermocycling‐ and fluorescence measurement‐related variabilities.

After evaluating the analytical detection specificity, sensitivity, and repeatability using well‐controlled biological samples, we lastly assessed the matrix effect of varying characterized clinical tissue and urine specimens on AVATAR analytical detection performance. To this end, we tested pilot cohorts of PCa patient tumor tissue (*n* = 35) (Figure 2e) and urine (*n* = 48) (Figure 2f) specimens to appraise the molecular analysis accuracy of AVATAR assay chemistry and device technology in different matrices. The clinical tumor and urine specimens were initially characterized by targeted RNA sequencing for T2:ERG, *PCA3*, *SChLAP1*, *HOXC6*, *DLX1*, *KLK2* biomarkers as an analytical gold standard methodology, for which the AVATAR analysis outcomes were subsequently compared against. Despite being challenged with RNA extracts from diverse patient tissue and urine sources, it was found that our AVATAR analysis has excellent correlation (mean Pearson's correlation coefficient = 0.94) in T2:ERG, *PCA3*, *SChLAP1*, *HOXC6*, *DLX1*, *KLK2* biomarker expression with targeted RNA sequencing (Figures S11 and S12). This dataset demonstrated the robustness of AVATAR despite different matrices of solid and liquid patient specimens, thereby supporting its feasibility for PCa clinical use.

### 2.3 Decentralized Clinical Urinary Specimen Analysis on Independent Training and Validation Cohorts

**FIGURE 3:**
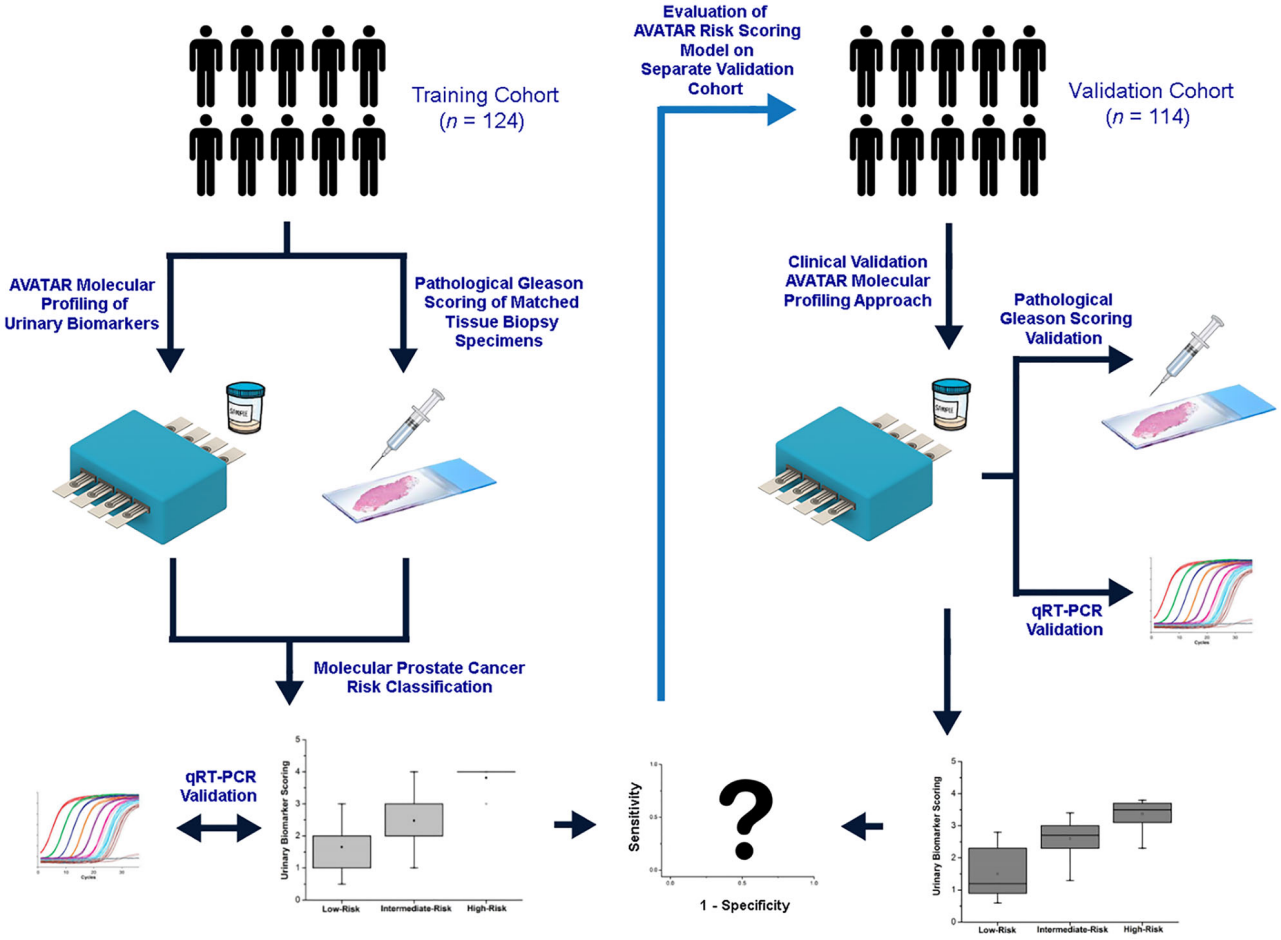
Clinical validation framework design for AVATAR technology using training and validation patient sample cohorts. A purposefully designed technology evaluation study encompassing independent well‐annotated training and validation cohorts of matched patient urinary and tissue biopsy specimens is used to clinically validate AVATAR's ability for prostate cancer diagnosis and risk stratification. A training patient cohort (*n* = 124) is first used to establish cross‐trained risk scoring model by correlating AVATAR urinary biomarker profiling to known patient biopsy outcomes for low‐, intermediate‐, and high‐risk prostate cancer subgroups. For more extensive clinical assessment of AVATAR, the risk scoring model is tested on a validation patient cohort (*n* = 114) to obtain clinical metrics. The validation cohort is similarly made up of matched patient urinary and tissue biopsy specimens. Using the risk scoring model, the patient specimens into different risk groups using AVATAR for comparison with patients’ biopsy outcomes. For both cohorts, qRT‐PCR is used as an orthogonal molecular validation method.

Beyond extensive analytical detection performance evaluation, the main obstacle hindering clinically translating novel biosensor technology research for liquid biopsy testing is the lack of comprehensive clinical technology validation studies to convincingly demonstrate and maximize clinical usefulness. To accomplish this, it is paramount for tailored studies to be designed from a clinical perspective with the inclusion of clinically accredited biomarkers and relevant cohorts of well‐annotated patient samples [18, 48]. Therefore, we purposefully designed a biomarker‐driven evaluation study encompassing independent well‐annotated training and validation cohorts of matched patient urinary and tissue biopsy specimens to clinically validate AVATAR's ability for decentralized PCa diagnosis, as well as PCa risk stratification (Figure 3).

**FIGURE 4:**
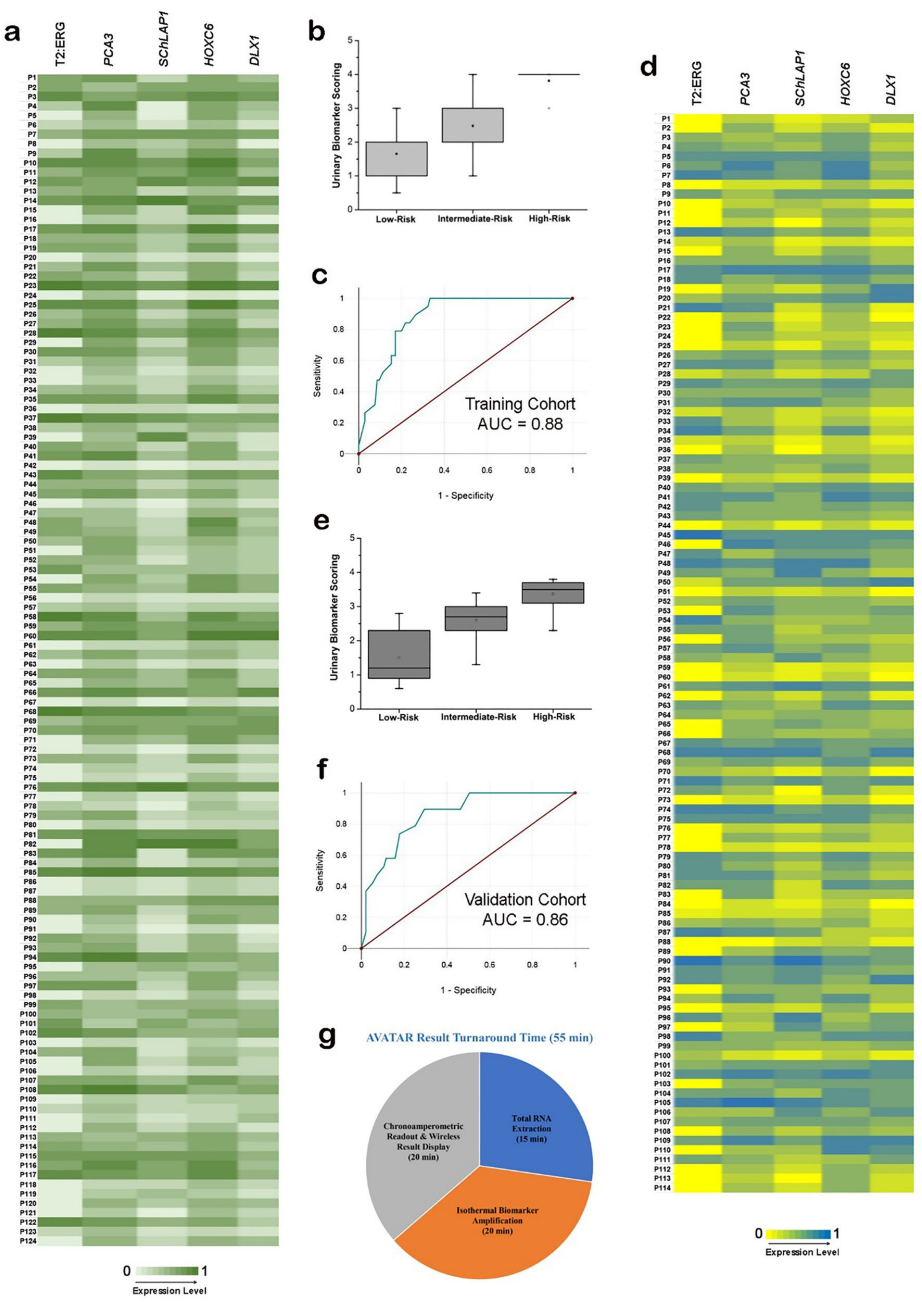
Decentralized AVATAR urinary biomarker profiling of independent training and validation patient cohorts. (a) Molecular profiling of prostate cancer biomarker panel (T2:ERG, *PCA3*, *SChLAP1*, *HOXC6*, *DLX1*, *KLK2*) in patient specimens. Clinical urinary and specimens were collected from individual prostate cancer patients (*n* = 124) and analyzed using AVATAR. (b) A risk scoring model is produced by relating AVATAR urinary biomarker expression levels to patient biopsy outcomes. The clinical specimens are grouped into low‐, intermediate‐, and high‐risk prostate cancer subgroups based on the pathological Gleason scoring of the matched biopsy tissue specimens. (c) Receiver operating characteristic plot of the risk scoring model on clinical urinary specimens used for training the model (AUC = 0.88) for high‐risk prostate cancer. (d) A separate validation cohort of patient specimens (*n* = 114) is used to clinically validate the AVATAR urinary biomarker profiling approach developed using the training cohort. (e) Using the risk scoring model, AVATAR urinary biomarker expression levels are sorted the patient specimens into low‐, intermediate‐, and high‐risk subgroups for comparison with patients’ biopsy outcomes. (f) Receiver operating characteristic plot of AVATAR's discriminatory ability for predicting high‐risk prostate cancer in the validation cohort (AUC = 0.86). (g) AVATAR provides a singular platform for streamlined assay workflow and immediate wireless result display within result turnaround time of 55 min. All signal measurements were performed in triplicate and normalized against *KLK2* expression level. The data are presented as mean values.

We first performed AVATAR for our nominated PCa urinary biomarker panel (T2:ERG, *PCA3*, *SChLAP1*, *HOXC6*, *DLX1*, *KLK2*) profiling on a training cohort (*n* = 124) that consisted of matched clinical urinary and tissue biopsy specimens (Table S3). As shown by the biomarker expression heatmap plot (Figure 4a), the biomarker expression levels of T2:ERG, *PCA3*, *SChLAP1*, *HOXC6*, and *DLX1* were normalized by *KLK2* expression level for each liquid biopsy sample, in order to account for varying amount of circulating RNA among the clinical specimens. *KLK2* expression level was used to normalize the expression levels of other biomarkers as it is a gene expressed primarily in prostate cells. Hence, it can serve as an ideal normalization control to correct for varying number of prostate cells in different patient samples, ensuring accurate comparisons of biomarker levels among the patient specimens [25]. We also performed qRT‐PCR (by a different operator to ensure unbiased analysis) as an orthogonal molecular validation method and attained good concordance between AVATAR and qRT‐PCR biomarker expression results via Passing and Bablok regression analysis (Figure S13).

Within this training cohort, we further grouped specimens into low‐, intermediate‐, and high‐risk PCa subgroups based on the pathological Gleason scoring of the matched biopsy tissue specimens according to an experienced uropathologist: low‐risk PCa was defined as Gleason score (GS) ≤ 6, intermediate‐risk PCa as GS = 7 (3 + 4 and 4 + 3), and high risk PCa as GS ≥ 8. Based on this classification, 64 (52%), 44 (35%), and 16 (13%) were respectively in the low‐, intermediate‐, and high‐risk PCa subgroups (Table 1). Using the AVATAR urinary biomarker expression levels of the patient training cohort, we also developed a cross‐trained risk scoring model based on linear regression to separate patients into the three low‐, intermediate‐, and high‐risk PCa subgroups based on molecular data (Figure 4b). It is observed that the detection signal overlapped for low‐ and intermediate‐risk patients due to the biomarker panel limitation in differentiating these two risk groups. Intermediate‐risk prostate cancer is generally considered to be in the grey zone and highly challenging to differentiate [49]. However, it is worth noting that our main aim in this work is to develop AVATAR toward discriminating high‐risk prostate cancer of clinical significance. In comparison to clinical gold standard pathology evaluation (Table S3) of matched tissue specimens, our AVATAR urinary biomarker profiling approach demonstrated high discriminatory ability for high‐risk (GS ≥ 8) PCa with an area‐under‐curve (AUC) of 0.88 (95% confidence interval: 0.84−0.92) in the training cohort (Figure 4c).

**TABLE 1:** Clinical characteristic summary of patients in training and validation cohorts. *IQR* interquartile range, *PSA* prostate specific antigen, *GS* Gleason score.

	Training Cohort	Validation Cohort
Samples (*n*)	124	114
Age (years) (mean (median:IQR))	78 (72: 63–85)	75 (73: 58–81)
Median PSA (ng/mL) (IQR)	4.9 (0.5‐16.0)	4.8 (0.5‐15.0)
Pathological Risk Classification		
GS≤ 6 (Low‐Risk)	64 (52%)	50 (44%)
GS = 7 (Intermediate‐Risk)	44 (35%)	47 (41%)
GS≥ 8 (High‐Risk)	16 (13%)	17 (15%)

For more extensive clinical assessment of AVATAR, we used a separate validation cohort of patient specimens (*n* = 114) obtained over a pre‐defined recruitment period to clinically validate the urinary biomarker profiling approach developed using the training cohort. Similar to the training cohort, the validation cohort was made up of matched patient urinary and tissue biopsy specimens (Table S4). We carried out the PCa biomarker panel profiling on the urinary specimens using AVATAR and applied the risk scoring model (Figure 4d). Using the risk scoring model, we first sorted the patient specimens into different risk groups for comparison with patients’ biopsy outcomes (Figure 4e). As before, for orthogonal molecular validation method, we used qRT‐PCR (by a different operator to ensure unbiased analysis) and attained good concordance between AVATAR and qRT‐PCR biomarker expression results via Passing and Bablok regression analysis (Figure S14). Based on tissue biopsy examination by an experienced uropathologist, the validation cohort consisted of 50 (44%), 47 (41%), and 17 (15%) in the low‐, intermediate‐, and high‐risk PCa subgroups, respectively (Table 1). Through comparisons with patients’ biopsy outcomes (Table S4) in this validation cohort, AVATAR achieved an AUC of 0.86 (95% confidence interval: 0.83−0.89) for predicting high‐risk (GS ≥ 8) PCa by molecular urinary biomarker profiling (Figure 4f). Compared to the current clinical PSA biomarker, which has an overall AUC of 0.60 to 0.70 for detecting PCa in diverse multiethnic cohorts [50] and has limitations in accurately identifying high‐risk PCa, our urinary biomarker panel and AVATAR technology demonstrated superior clinical performance and could significantly reduce unnecessary prostate biopsies.

Crucially, through the extensive analysis of clinical urinary specimens in both training and validation cohorts, we found that our AVATAR molecular profiling approach significantly streamlined the result turnaround time. In contrast to a conventional workflow whereby a urinary specimen must be sent to a centralized laboratory for qRT‐PCR profiling of the urinary RNA biomarkers prior to test results being sent back to the treating clinician, AVATAR provides a singular platform for expedited assay workflow and immediate wireless result display within 55 min of sample collection (Figure 4g). To this end, the isothermal biomarker amplification and miniaturized chronoamperometric readout on our AVATAR platform facilitates rapid and decentralized molecular PCa risk stratification for expected patient benefit.

### 2.4 Molecular Correlation of Matched Patient Liquid Biopsy and Tissue Specimens to Clinically Assess AVATAR Technology

**FIGURE 5:**
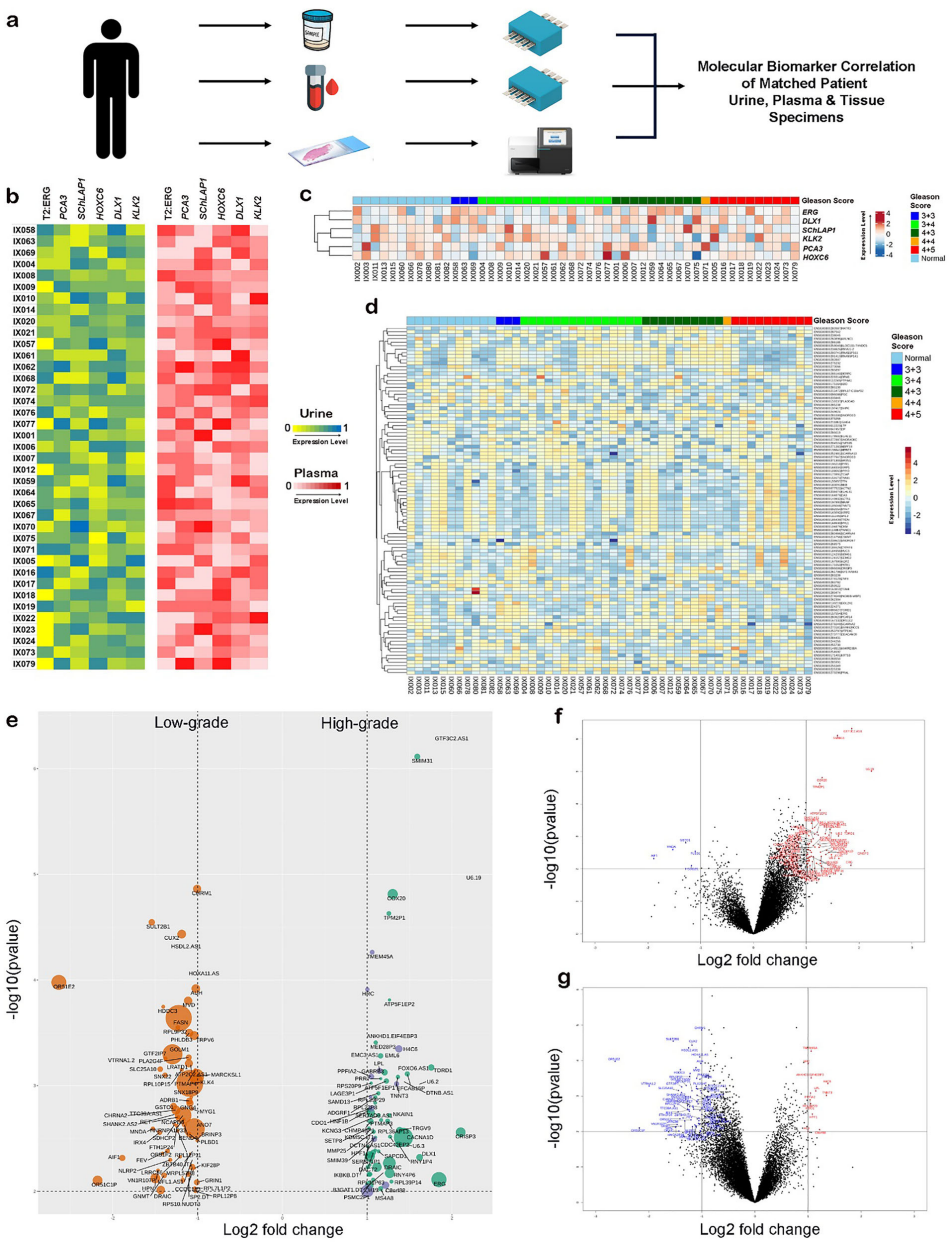
Molecular prostate cancer biomarker correlation between matched patient liquid biopsy and solid tissue specimens. (a) A tailored multi‐year PCa follow‐up study in an Australian patient cohort is carried out to track a cohort of men who have been diagnosed with prostate cancer and eventually proceeded on to radical prostatectomy (RP). Matched pre‐RP liquid biopsy urine and plasma (*n* = 39), as well as RP tissue specimens (*n* = 50, 11 including non‐cancerous tissues as controls), are collected. For biomarker correlation studies, AVATAR molecular profiling was performed on urinary and plasma specimens, and whole comprehensive transcriptomic sequencing was performed on pathologically identified cancerous foci of RP tissue specimens. (b) AVATAR biomarker (T2:ERG, *PCA3*, *SChLAP1*, *HOXC6*, *DLX1*, *KLK2*) profiling data between matched patient urinary (Pearson's correlation coefficient = 0.91) and plasma (Pearson's correlation coefficient = 0.82) are found to be concordant with matched tissue biomarker expression levels. All signal measurements were performed in triplicate and normalized against *KLK2* expression level to presented as a heat map. The data are presented as mean values. (c) Expression heatmap for all six genes of interest (*ERG*, *PCA3*, *SChLAP1*, *HOXC6*, *DLX1*, *KLK2*) based on whole transcriptomic sequencing of RP tissue specimens. Headers show tissue specimen classification into normal controls, low‐risk, favorable and unfavorable intermediate‐risk and high‐risk prostate cancer subgroups based on the Gleason scoring. (d) Expression heatmap of the 100 variable (over‐/under‐expressed) genes based on whole transcriptomic sequencing analysis across the RP tissue specimens classified into different prostate cancer risk groups. (e) Bubble volcano plot showing differentially expressed genes between two clinically relevant prostate cancer risk groups—by Gleason score (GS)—of high‐grade (GS 4+3 and above) versus low‐grade (healthy controls and GS 3+4 and below). (f,g) Volcano plots showing differentially expressed genes between GS 4+3 and GS 4+4 and above (red; right) versus low‐grade (blue; left) prostate cancer.

To validate and support the patient urinary specimen AVATAR findings from both clinical feasibility and biological contexts, we sought to study the molecular PCa biomarker correlation between matched patient liquid biopsy and solid tissue specimens (i.e., circulating liquid PCa biomarkers versus mutations in primary source of PCa). Consequently, we initiated a tailored multi‐year PCa follow‐up study in an Australian patient cohort to robustly evaluate AVATAR‐based PCa liquid biopsy testing as a viable clinical alternative to tissue biopsy biomarker profiling. In this study, we tracked a cohort of men who have been diagnosed with PCa and eventually proceeded on to radical prostatectomy (RP, surgical removal of prostate gland) treatment. We collected matched pre‐RP liquid biopsy urine and plasma, as well as RP tissue specimens from each individual patient for molecular profiling. It is noteworthy that we decided on the use of RP tissues specimens to provide a more comprehensive assessment of tumor pathology and sequencing input material as compared to core needle biopsy samples. The liquid biopsy urinary and plasma specimens were analyzed using AVATAR, and whole comprehensive transcriptomic sequencing was performed on pathologically identified cancerous foci of RP tissue specimens (i.e., origin of PCa tumor cells) (Figure 5a).

We completed whole transcriptomic sequencing on 50 RP tissue specimens, including 39 specimens with matched urinary and plasma counterparts and 11 non‐cancerous tissues as controls. We first filtered out to sequencing data to evaluate the expression levels of our six urinary biomarker panel (T2:ERG, *PCA3*, *SChLAP1*, *HOXC6*, *DLX1*, *KLK2*). For T2:ERG, we opted to directly assess the levels of *ERG* since this PCa gene fusion mutation will eventually result in *ERG* overexpression. We classified the 50 RP tissue specimens into normal controls, low‐risk GS 6, intermediate‐risk GS 7 (3+4, 4+3), high‐risk GS 8, and GS 9; of which 3+4 and 4+3 are additionally classified as favorable and unfavorable, respectively. We found that the AVATAR biomarker profiling data (Figure 5b) of matched patient urinary (mean Pearson's correlation coefficient = 0.91) (Figure S15) and plasma (mean Pearson's correlation coefficient = 0.82) (Figure S16) correlated well with matched tissue biomarker expression levels (Figure 5c). The overall concordance of our six PCa biomarker panel expression between matched tumor tissue and liquid biopsies supports the use of liquid biopsy as a viable alternative to invasive surgical biopsy procedures. This finding agrees well with several prominent studies on molecular mutation profiling of paired solid and liquid biopsies from various cancer types. Interestingly, the urinary specimens displayed a higher concordance to matched tissue specimens than plasma specimens, possibly due to the anatomical position of the prostate (tumor growth origin) being connected to the urethra for direct shedding of tumor cells. Coupled with the potential circulating tumor RNA concentration effect by the kidneys via renal filtration, these factors enable urinary sampling to be an ideal source of PCa liquid biopsy as compared to low tumor content in small volumes of blood sampling.

Given the range of different biological cancer pathways driving PCa in individuals resulting in clinical and molecular heterogeneities [51, 52, 53], accurate determination of PCa recurrence after primary treatment will most probably require a wide multiplex panel of molecular biomarkers. While our six‐RNA biomarker panel on AVATAR has shown superior clinical performance over current PSA testing for discriminating high‐risk PCa, it is difficult to conceive that the early detection of eventual aggressive PCa subtypes would be limited to few markers early in the disease course. To further investigate PCa RNA biomarker candidates and high‐grade PCa‐associated biological pathway in this Australian patient cohort to improve risk stratification on AVATAR, we then leveraged the rich whole transcriptomic sequencing dataset to uncover the top variable (over‐/under‐expressed) transcripts to correlate with various pathological Gleason scoring categories. We identified the top 100 variable transcripts associated with the different risk classification groups (Figure 5d), revealing the high molecular heterogeneity of the PCa transcriptomic landscape. Using bioinformatics analysis to sort the top‐ranked transcripts (Figure 5e) into two clinically relevant PCa risk groups of high‐grade (GS 4+3 and above) vs low‐grade (healthy controls and GS 3+4 and below), we obtained two separate sets of transcripts to differentiate the different groups (Figure 5f,g). Furthermore, pathway enrichment analysis showed the muscle contraction signaling (involving *ACTA1*, *ACTN2*, *MYH3*, *MYL1*, *MYL2*, *NEB*, *TCAP*, *TNNC1*, *TNNI1*, *TNNT1*, *TTN*) as the top high‐grade PCa‐associated pathway. This is supported by recent similar findings [54, 55, 56] of hub genes mainly involved in muscle contraction being significantly enriched in PCa with higher GS and poorer prognosis. We reasoned that a possible mechanism for this impacted biological pathway is that muscle contraction can promote tumor dissemination and metastasis around the body by increasing luminal pressure and promotingprostate cancer cell invasion into the lymphatic vessels [57, 58, 59], thus leading to high‐grade metastatic PCa. Our analysis suggested that one or several genes of the muscle contraction signaling may be incorporated as new biomarkers on AVATAR to improve PCa risk stratification in a rapid, decentralized approach.

## 3 Discussion

Biosensor technology is a rapidly evolving research domain that holds immense promise for decentralized liquid biopsy applications. Despite the multitude of innovations in liquid biopsy technologies in recent times, a scarce number of biosensor platforms have been clinically translated, primarily due to inadequate clinical validation. In this work, we strived to address this deficit by executing a research pipeline of novel biosensor (AVATAR) platform technology development, followed by rigorous validation studies into its clinical performance and feasibility for PCa urinary risk stratification and molecular profiling correlation with matched patient plasma and tissue specimens under a clinical translation framework.

AVATAR is a miniaturized biosensor technology that enables multiple PCa circulating tumor RNA biomarker profiling and real‐time wireless smartphone result communication in a rapid (55 min) and decentralized manner. Miniaturized devices that similarly utilized magnetic nanoparticles to facilitate chronoamperometric biomarker detection with smartphone pairing have been previously reported. These include an eight‐channel device for exosomal protein detection in cancer [60], an eight‐channel device for antigen detection in food allergies [61], and a single‐channel device for cytokine detection in sepsis [62]. In comparison, AVATAR provides equivalent advantages in a palm‐sized equipment footprint, simultaneous multi‐channel detection capacity and smartphone interfacing capability; and AVATAR possesses the unique technical benefit of being compatible with an assay chemistry for detecting multiple clinically validated RNA biomarkers in prostate cancer liquid biopsies.

Together with its customized isothermal assay chemistry, AVATAR has demonstrated excellent analytical single cell‐level detection capabilities for clinically relevant levels of specific PCa urinary biomarkers. Importantly, the short sample‐to‐result turnaround time and portability of AVATAR can reduce the logistics cost of storing and sending a patient specimen to a centralized laboratory and shorten the period of patient anxiety while awaiting testing outcomes. Furthermore, having access to quicker and accurate molecular testing results will enable earlier completion of disease diagnosis and guidance of the treatment plan, thus driving better patient outcomes.

As compared to centralized testing of circulating RNA biomarkers in liquid biopsies, AVATAR can facilitate decentralized testing by substantially reducing the logistical reliance on sophisticated laboratory‐based instruments. Isothermal RPA can be performed with a heat block at a single temperature without a thermocycler, magnetic processing can be achieved with a small magnetic plate without a centrifuge machine, and electrochemical readout can be accomplished with a miniaturized potentiostat (i.e. AVATAR device) instead of complex wavelength filtering systems for conventional fluorescence readout. Although the current AVATAR workflow still requires a certain degree of technical specimen handling similar to point‐of‐need COVID‐19 rapid antigen testing, we rationalize that the increased isothermal assay robustness and reduced hands‐on time is more feasible for on‐site analysis (as compared to mixing light‐sensitive fluorescence dyes for conventional qRT‐PCR analysis). Moreover, future workflow automation through RPA reagent lyophilization and magnetic processing robotics is possible to negate the need of specimen handling.

Since AVATAR is designed for rapid decentralized molecular profiling of a targeted PCa biomarker panel, its current eight‐sample throughput is equivalent to qRT‐PCR but significantly lower than targeted RNA sequencing (tens to hundreds‐sample throughput). Thus, it is envisaged that AVATAR is best suited for a smaller disease‐specific biomarker panel (as showcased for PCa in this study), which could provide clinically useful information in a decentralized setting. Nonetheless, it is worthy to highlight that the electrochemical readout of AVATAR is highly scalable and has the feasible potential for higher sample throughput.

In this study, we aimed to address the lack of extensive clinical validation of innovative decentralized liquid biopsy biosensor technologies, which can hamper successful clinical translation. To this end, we purposefully designed the clinical validation of AVATAR technology to include 1) a panel of clinically validated PCa urinary biomarkers for testing; 2) detection outcome comparisons against both molecular (qRT‐PCR) and pathological (Gleason scoring) gold standard methodologies; 3) the use of independent training (*n* = 124) and validation (*n* = 114) cohorts. These experimental conditions ensure that AVATAR can be robustly evaluated for non‐invasive PCa risk stratification. We found that the eventual AUC of AVATAR for discriminating high‐risk PCa cases is 0.86. Our achieved AUC of 0.86 for discriminating high‐risk prostate cancer is similar/potentially higher to various established prostate cancer urine tests such as MyProstateScore (AUC = 0.73) [25]; SelectMDx (AUC = 0.85) [30]; and especially an up‐to‐date new laboratory‐based urinary test (MPS2) incorporating 18 biomarkers overexpressed by high‐grade PCa (AUC = 0.81) [63]. While acknowledging that differences in patient cohorts and assay techniques limit direct comparisons, it is evident that AVATAR could significantly improve traditional PSA‐based testing. Combined with the technological and assay chemistry aspects of AVATAR in enabling rapid decentralized liquid biopsy molecular profiling, our clinical validation results solidify the PCa clinical application on our platform for patient benefit.

We extensively evaluated the clinical feasibility of biosensor‐driven PCa liquid biopsies as an alternative to invasive tissue biopsies by collecting 39 matched urinary, plasma and RP tissue specimens for molecular analyses. It is worth highlighting that, unlike the majority of previous bulk biobank studies, this clinical study is tailored for the multi‐year precision disease progression tracking of Australian PCa patients from initial PCa diagnosis to RP treatment. This allowed for consistent matched molecular liquid biopsy and tissue characterization of the transcriptomic landscape at an equivalent clinical timepoint of PCa progression for all PCa patients (i.e., proceeding to RP after unfavourable pathological biopsy outcome). Our data supported the clinical use of liquid biopsy biomarker profiling via AVATAR with statistically significant agreement to matched tissue biomarker expression (mean Pearson's correlation coefficient = 0.91) [44, 64]. This encouraging outcome also supports a potential future application of AVATAR as a precision cancer monitoring technology by using a patient's solid tumor tissue specimens to design a personalized liquid biopsy biomarker panel (unique to the individual's cancer mutation profile), which can be implemented on AVATAR to early detect any post‐treatment disease recurrence [65].

By leveraging the whole transcriptomic sequencing datasets of this Australian patient cohort, we gained additional biological and clinical insights into the association of muscle contraction signaling with high‐grade PCa. This warrants further investigation of the genes within this biological pathway as novel PCa early diagnosis and risk stratification biomarkers for use on AVATAR. This is an interesting observation for reporting as it is consistent with independent international studies, which have similarly reported muscle contraction being significantly enriched in prostate cancer with higher Gleason scoring and poorer prognosis [54, 55, 56]. However, we acknowledged that further validation experiments are required before these prospective biomarkers can be included in our current urinary biomarker panel.

Taken together, AVATAR represents a PCa biosensor technology realized through multidisciplinary fields of biological, chemical, engineering, computational, and clinical sciences. AVATAR enables rapid, accurate, and decentralized molecular profiling of PCa liquid biopsy specimens with analytical and clinical performances that were robustly validated via matched tissue and liquid biopsy patient specimens from a unique multi‐year PCa tracking study. AVATAR underlines the use of powerful liquid biopsy profiling technology to gain a comprehensive molecular snapshot for PCa early detection and risk stratification. Furthermore, in the PCa treatment setting, AVATAR could offer decentralized companion diagnostic testing of predictive biomarkers to modern precision PCa therapeutics such as radioligand therapies [66]. Whilst this study focuses on PCa, the use of AVATAR is broadly applicable to nucleic acid biomarkers of diverse diseases.

## 4 Methods

### 4.1 Cell Culture

PCa cancer cell lines: DuCap (Research Resource Identifier (RRID):CVCL_2025), LnCap (RRID:CVCL_0395), and 22Rv1 (RRID:CVCL_1045) were obtained from the American Type Culture Collection (ATCC). All cell lines were tested and free of mycoplasma contamination. The PCa cell lines were cultured in RPMI‐1640 growth media (Life Technologies) supplemented with 10% fetal bovine serum (Life Technologies) and kept in a humidified incubator containing 5% CO_2_ at 37°C.

### 4.2 Clinical Specimens

For clinical urine, blood, and tissue specimens, ethics approval was obtained from The University of Queensland Institutional Human Research Ethics Committee (Approval No. 2004000047), and Royal Brisbane & Women's Hospital Human Research Ethics Committee (Ref No. 1995/088B). Informed consent was obtained from all subjects prior to specimen collection, and methods pertaining to clinical specimens were carried out in accordance with approved guidelines.

### 4.3 Total RNA Extraction

Total RNA was extracted from PCa cells lines, urine, and plasma samples using Direct‐zol RNA MiniPrep Kit (Zymo Research), ZR Urine RNA Isolation Kit (Zymo Research), and Quick‐cfRNA Serum and Plasma Kit (Zymo Research), respectively, according to manufacturer's instructions. Total RNA was extracted from clinical FFPE tissue samples using AllPrep DNA/RNA FFPE Kit (Qiagen) according to manufacturer's instructions. Total RNA in the urine specimens was extracted using the commercially available ZR urine RNA isolation Kit (Zymo Research). Briefly, urinary cells were enriched by passing 30 mL of urine, followed by 700 µL of supplied urine RNA buffer through the supplied ZRC GF Filter. Collected urinary cells were then lyzed, washed, and eluted in 10 µL of RNase‐free water according to manufacturer's instructions. 1.5 µL of extracted RNA was used for RPA. Cell‐free RNA was extracted from 2 mL of plasma specimen using the Zymo Quick‐cfDNA/cfRNA Serum and Plasma Kit (Zymo Research). Large cell debris was first removed by centrifugation. The plasma proteins were digested by proteinase K (40U) for 2 h at 37°C. 100% isopropanol was added (1.5 volumes), and the entire solution vacuum filtered through a spin column for cell‐free nucleic acid binding. Cell‐free RNA was selectively isolated, purified by 100% ethanol precipitation, and eluted in 10 µL of RNase‐free water according to manufacturer's instructions. 1.5 µL of extracted RNA was used for RPA. The extracted total RNA was quantified using Qubit RNA High Sensitivity Assay Kit (Thermo Fisher Scientific).

### 4.4 Fabrication of the Miniaturized AVATAR Device

The AVATAR device consists of a microcontroller (ATSAMD21G18 ARM Cortex M0 processor, Microchip Technology Inc.) onboard an off‐the‐shelf module (Adafruit Feather M0 Bluefruit LE, Adafruit Industries LLC), a digital‐to‐analog converter (DAC8552, Texas Instruments), an analog‐to‐digital converter (ADC7684, Analog Devices), a multiplexer (ADG708, Analog Devices), and eight potentiostats. Each potentiostat consists of two operational amplifiers (AD8606, Analog Devices): one amplifier maintains the potential difference between a working electrode and a reference electrode, and the other one works as a transimpedance amplifier to convert a current to a voltage signal. The current measuring range of the transimpedance amplifier was ±7.5 µA. The eight‐channel electrodes are commercially available (Metrohm DropSens). The firmware on the microcontroller collects the digitized measurement data, adds data labels and additional user interface text, and sends it in human‐readable text format over Bluetooth virtual serial port. The mobile app is based on a ready‐to‐use app called “Serial Bluetooth Terminal” which we customized for use with AVATAR. The app is designed to connect to devices and interact with them via sending and receiving serial text commands and data. It can save the serial text data into a text file, which can then be sent to a laptop or other device via Bluetooth. The app is free, open source, and available for unrestricted use, copy, modification, merge, publish, distribute, sublicense, and/or sell under the Massachusetts Institute of Technology (MIT) license.

### 4.5 Surface Functionalization of Magnetic Beads

Primer‐functionalized magnetic nanoparticles were individually prepared for each PCa RNA biomarker by incubating 100 µL of 10 nm Absolute Mag Streptavidin Iron Oxide Nanoparticles (CD Bioparticles) with 100 µL of 10 µm corresponding biotin‐modified forward primer sequences (IDT; Table S1) on a mixer at room temperature for 30 min. After surface functionalization, excess unbound primers were removed using magnetic washing with Wash/Storage Buffer (CD Bioparticles), and primer‐functionalized magnetic nanoparticles were resuspended in 200 µL of UltraPure DNase/RNase‐free water (Invitrogen).

### 4.6 AVATAR Assay

To carry out isothermal recombinase polymerase amplification (RPA) on the forward primer‐functionalized surfaces of magnetic nanoparticles, the TwistAmp Basic RT kit (Twist‐DX) was used with slight modifications to manufacturer's instructions. 1.5 µL of extracted RNA, 375 nm of reverse primer (IDT; Table S1), 20 nm of biotin‐modified uracil bases (Thermo Fisher Scientific), and 5 µL associated set of corresponding forward primer‐functionalized magnetic beads were added to make a 12.5 µL reaction volume prior to incubation at 41°C for 20 min. After isothermal RPA on magnetic nanoparticles, amplicons for each PCa RNA biomarker were magnetically washed with Wash/Storage Buffer (Creative Diagnostics), incubated with 1 µL 1:1000 diluted of 1.25 mg/mL streptavidin–horseradish peroxidase (HRP; Thermo Fisher Scientific) in 10 mm phosphate‐buffered saline (Thermo Fisher Scientific) with 0.5% Triton X‐100 (Sigma–Aldrich) for 5 min to label biotin‐modified uracil bases within the amplicons. The amplicons were washed again before being magnetically loaded onto the working electrode surface of screen‐printed electrode (DRP‐C110‐U75, Metrohm Dropsens) by positioning a permanent magnet under the electrode. 15 µL of 1‐Step TMB (3,3′,5,5′‐tetramethylbenzidine) solution (Thermo Fisher Scientific) was added to the surface‐loaded magnetic nanoparticles before 500 mM of H_2_SO_4_ (Sigma–Aldrich) was added after 5 min to stop the reaction and activate TMB for chronoamperometric measurements. Each screen‐printed electrode was inserted into the AVATAR device for chronoamperometric measurement at 150 mV for 30 s. All measurements were performed at room temperature using a custom‐built mobile app.

### 4.7 Risk Scoring Model Development

For developing the risk‐scoring model, we used AVATAR measurements from low‐, intermediate‐, and high‐risk prostate cancer subgroups (based on Gleason scoring) in the patient training cohort. We recorded the AVATAR measurements as the predictor variables, and categorical risk status (low‐/intermediate‐ vs high‐risk) as the outcome variable in linear regression. To determine the model's performance and avoid overfitting, leave‐one‐out cross‐validation was performed. The averaged regression coefficients from the cross‐validation were used to create a linear regression model. We applied and validated this regression model for scoring all clinical samples (training and validation cohorts).

### 4.8 Real‐Time Reverse Transcription Polymerase Chain Reaction (qRT‐PCR) Analysis

The KAPA SYBR FAST One‐Step qRT‐PCR kit (KAPA Biosystems) was used to set up a single 10 µL reaction volume for each sample. Each reaction volume consisted of 1X KAPA SYBR FAST qPCR Master Mix, 200 nm of forward and reverse primers (IDT; Table S1), 1X KAPA RT Mix, 50 nm of ROX dye, and 3 µL of extracted RNA. qRT‐PCR was performed using the Applied Biosystems 7500 Real‐Time PCR System (Thermo Fisher Scientific). The cycling protocol was: 42°C for 10 min to synthesize cDNA, followed by 95°C for 5 min before cycling 40 times (95°C for 30 s, 50°C for 30 s, and 72°C for 1 min), and finished with 72°C for 10 min.

### 4.9 Whole Transcriptomic Sequencing Analysis

For strand‐specific RNA sequencing analysis of clinical tissue specimens, the whole transcriptome libraries were prepared using the Collibri Stranded RNA Library Prep Kit with Collibri rRNA Depletion Kit (Invitrogen). The extracted total RNA from clinical FFPE tissue specimens was first concentrated using AMPure XP beads (Beckman Coulter) and assessed for quality and size distribution using the RNA 6000 Pico Assay (Agilent). Then, rRNA‐depleted RNA was prepared from the total RNA samples using the Collibri rRNA Depletion Kit (Invitrogen) to specifically remove ribosomal RNA by using a set of affinity probes. Following rRNA depletion, the quality and size distribution of the rRNA‐depleted samples were again analyzed using the Agilent RNA 6000 Pico Assay (Agilent) before proceeding to library preparation and sequencing. To prepare the sequencing libraries, RNA was fragmented using RNase III, and the fragmented RNA was purified using Dynabeads Cleanup Beads (Invitrogen). Next, adaptors were hybridized and ligated to the fragmented and purified RNA before reverse transcription was performed on the adaptor‐ligated RNA mixture to generate cDNA. Thereafter, the cDNA was purified using Dynabeads Cleanup Beads (Invitrogen) prior to indexing PCR. The amplified cDNA was purified using Dynabeads Cleanup Beads (Invitrogen), and the resultant prepared libraries were assessed for yield and size distribution using the High Sensitivity DNA Kit (Agilent). Lastly, the prepared libraries were denatured, diluted, and loaded for sequencing on the NextSeq500 System (Illumina) according to manufacturer's protocol.

### 4.10 Bioinformatics Analysis

Transcriptome paired‐end sequencing reads of 100 bp were generated using a NextSeq500 instrument to a targeted depth of 100 million reads per sample. Adapter sequences were removed using Cutadapt (v1.11) and aligned using STAR (v2.5.2a) to the GRCh37 assembly and Ensembl v75 gene model [67]. Quality control metrics were computed using RNA‐SeQC (version 1.1.8). Gene expression quantified as TPM (transcripts per million), and gene counts were estimated using RSEM (v1.2.30) [68]. For differential expression analysis, clinical samples were categorized into benign and aggressive based on their Gleason scores: normal and samples with primary + secondary Gleason Scoring 3+3 and 3+4 were assigned to ‘low‐grade’, whereas 4+3, 4+4, 4+5, and 5+4 assigned ‘high‐grade’. Differentially expressed (DE) genes between low‐grade vs high‐grade were calculated using DESeq2 [69]. The significant DE genes (FDR< 0.05) were visualized as volcano plots and heatmaps using ggplot2 and pheatmap R packages, respectively. For pathway analysis, the significantly differentially expressed genes were mapped to known pathways from KEGG and Reactome using the InnateDB tool to identify significantly enriched pathways among these differential genes.

### 4.11 Statistical Analyses

All measurements were performed in triplicate, and the data were displayed as means ± standard deviation, unless otherwise stated. Correlation analysis was performed with linear regression to determine the goodness‐of‐fit (R^2^), unless otherwise stated. Statistical analyses were performed using OriginLab (v.10.0). For clinical analysis, sample sizes were restricted by the availability of patient samples. No data were excluded from the analyses. All experiments were performed blinded from the clinical outcomes.

## Author Contributions

K.M.K. and G.P. contributed equally to this work. K.M.K., P.N.M., R.A.G., and M.T. designed the study. K.M.K., G.P., S.S., and M.J.R. wrote the manuscript. K.M.K., G.P., S.S., B.J., K.J.F., K.‐L.R., and D.J.K. performed the research and data analysis. Á.F., J.W.Y., H.S., and S.A.T. provided clinical samples. H.S. provided pathology evaluation.

## Conflicts of Interest

The authors declare no competing financial interest. K.M.K., G.P., B.J., K.J.F. were employees, and P.N.M. and M.T. were cofounders, of XING Technologies Pty Ltd. S.A.T. is a cofounder of Strata Oncology.

## Supporting information




**Supporting File 1**: advs73408‐sup‐0001‐SuppMat.docx.

**Supporting File 2**: advs73408‐sup‐0002‐VideoS1.mp4.

## Data Availability

The data that support the findings of this study are available from the corresponding author upon reasonable request.
