# Supplementary material for: Rapid Decentralized Prostate Cancer Risk Stratification by Portable Liquid Biopsy Analysis within a Clinical Biosensor Validation Framework
Source: Adv Sci (Weinh). 2026 Jan 27;13(13):e12126. doi: 10.1002/advs.202512126 (PMC12955865; doi:10.1002/advs.202512126)
Supplement: Supplementary file 1 — Supporting File 1: advs73408‐sup‐0001‐SuppMat.docx. [file ADVS-13-e12126-s001.docx]

**SUPPLEMENTARY INFORMATION**

**Rapid decentralized prostate cancer risk stratification by portable liquid biopsy analysis within a clinical biosensor validation framework**

Kevin M. Koo^1,2,3,12*^, Grant Phillips^2,12^, Sriganesh Srihari^4^, Áine Farrell^1^, Binny Jaradi^2^, Kira J. Fitzpatrick^2^, John W. Yaxley^1,5^, Hemamali Samaratunga^1,6,7^, Paul N. Mainwaring^2^, Ke-lin Ru^3^, Darren J. Korbie^3^, Scott A. Tomlins^8,9^, Matthew J. Roberts^1,5,7*^, Robert A. Gardiner^1,5,10*^ and Matt Trau^3,11*^

^1^The University of Queensland Centre for Clinical Research (UQCCR), Brisbane, QLD, Australia.

^2^XING Applied Research & Assay Development (XARAD) Division, XING Technologies Pty Ltd, Brisbane, QLD, Australia.

^3^Centre for Personalized Nanomedicine, Australian Institute for Bioengineering and Nanotechnology (AIBN), The University of Queensland, Brisbane, QLD, Australia.

^4^QIMR Berghofer Centre for Immunotherapy and Vaccine Development and Translational and Human Immunology Laboratory, Department of Immunology, QIMR Berghofer Medical Research Institute, Brisbane, QLD, Australia.

^5^Department of Urology, Royal Brisbane and Women’s Hospital, Brisbane, QLD, Australia.

^6^Aquesta Specialized Uropathology, Brisbane, QLD, Australia.

^7^Faculty of Health, Medicine and Behavioural Sciences, The University of Queensland, Brisbane, QLD, Australia.

^8^Department of Pathology, University of Michigan Medical School, Ann Arbor, MI, USA

^9^Strata Oncology, Ann Arbor, MI, USA

^10^Edith Cowan University, Perth, WA, Australia.

^11^School of Chemistry and Molecular Biosciences, The University of Queensland, Brisbane, QLD, Australia.

^12^These authors contributed equally.

Corresponding Authors:

maisheng.koo@uqconnect.edu.au;

m.roberts2@uq.edu.au;

f.gardiner@uq.edu.au;

m.trau@uq.edu.au

**CONTENTS**

**Supplementary Figure 1.** Design of the AVATAR device.

**Supplementary Figure 2.** Specific isothermal amplification of biomarkers.

**Supplementary Figure 3.** Chronoamperometric signals corresponding to biomarker expressions levels in the three well-characterized prostate cancer cell lines (DuCap, LnCap, 22Rv1).

**Supplementary Figure 4.** Validation of biomarker expressions levels in the three well-characterized prostate cancer cell lines (DuCap, LnCap, 22Rv1).

**Supplementary Figure 5.** Optimization of primer concentrations (300, 325, 350, 375, 400 nM).

**Supplementary Figure 6.** Optimization of biotin-modified uracil base concentrations (10, 15, 20, 25, 30 nM).

**Supplementary Figure 7.** Optimization of magnetic bead volumes (1.2, 2.5, 5.0, 7.5, 10.0 µL).

**Supplementary Figure 8.** Optimization of isothermal amplification time (0, 5, 10, 15, 20, 25 min).

**Supplementary Figure 9.** Optimization of TMB (3,3′,5,5′-tetramethylbenzidine) incubation time (1, 3, 5, 7 min).

**Supplementary Figure 10.** Optimization of voltage for chronoamperometric readout (50, 100, 150, 200 mV).

**Supplementary Figure 11.** Scatter plots per gene between AVATAR and targeted RNA-sequencing measurements in clinical tissue specimens.

**Supplementary Figure 12.** Scatter plots per gene between AVATAR and targeted RNA-sequencing measurements in clinical urinary specimens.

**Supplementary Figure 13.** Passing−Boblok regression analysis of the measurements from AVATAR versus qRT-PCR in the training cohort.

**Supplementary Figure 14.** Passing−Boblok regression analysis of the measurements from AVATAR versus qRT-PCR in the validation cohort.

**Supplementary Figure 15.** Scatter plots per gene between AVATAR and whole transcriptomic sequencing measurements in clinical urinary specimens.

**Supplementary Figure 16.** Scatter plots per gene between AVATAR and whole transcriptomic sequencing measurements in clinical plasma specimens.

**Supplementary Table 1.** Primer sequences used in experiments.

**Supplementary Table 2.** Comparisons of AVATAR with recently reported isothermal electrical signaling-based assays for circulating RNA detection in clinical cancer specimens.

**Supplementary Table 3.** Training cohort (*n* = 124). Tumor Gleason scores for patient biopsy samples and AVATAR biomarker expression levels.

**Supplementary Table 4.** Validation cohort (*n* = 114). Tumor Gleason scores for patient biopsy samples and AVATAR biomarker expression levels.

**
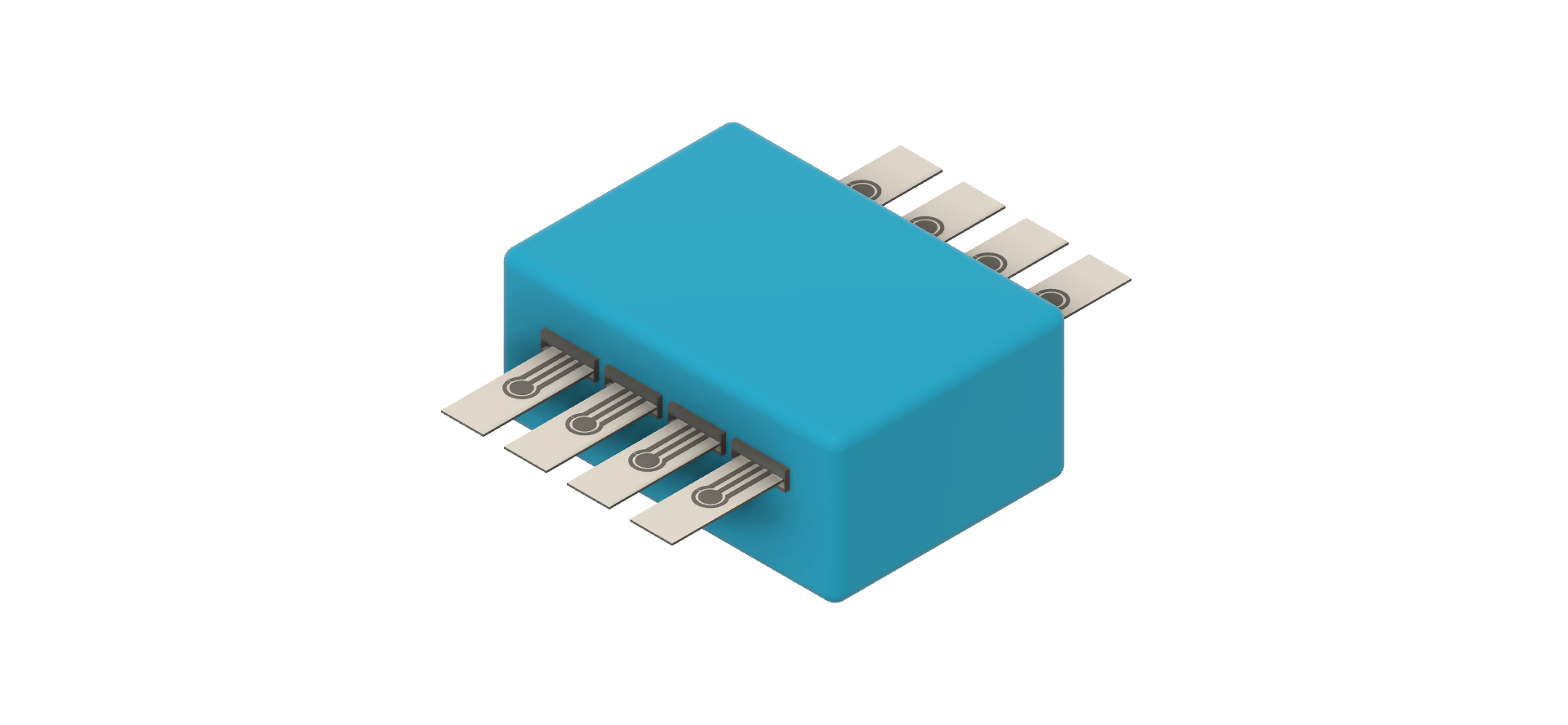
**
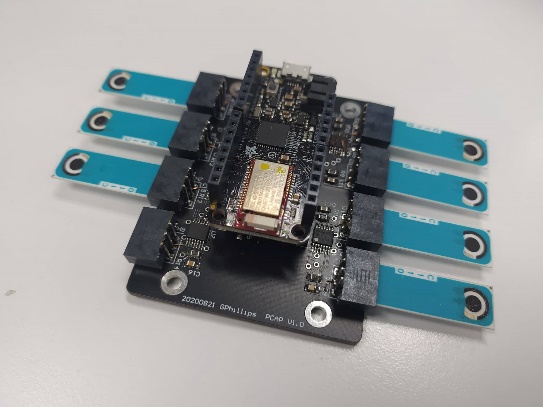


**Supplementary Figure 1. Design of the AVATAR device.** AVATAR is constructed to be of palm-sized equipment footprint with eight separate slots for insertion and output signal measurement of individual assays on independent gold electrode surfaces.

**
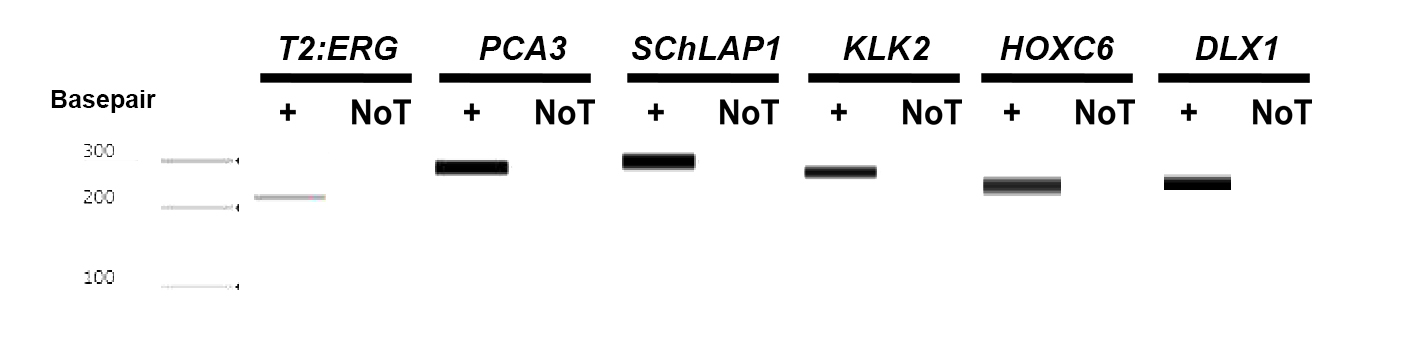
**

**Supplementary Figure 2.** **Specific isothermal amplification of biomarkers.** Gel electrophoresis of T2:ERG (216 bp), *PCA3* (281 bp), *SChLAP1* (282 bp), *HOXC6* (239 bp), *DLX1* (244 bp), *KLK2* (256 bp) amplicons with expected base pair sizes.


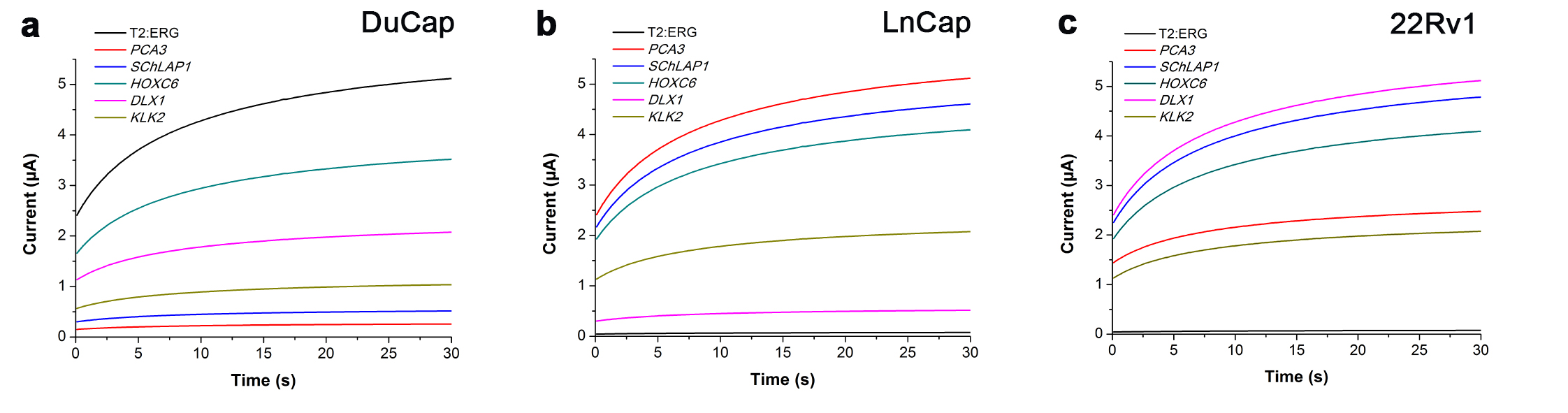


**Supplementary Figure 3. Chronoamperometric signals corresponding to biomarker expressions levels in the three well-characterized prostate cancer cell lines (DuCap, LnCap, 22Rv1).** AVATAR provided quantitative measurements of different biomarker levels in human prostate cancer cell-derived RNA.


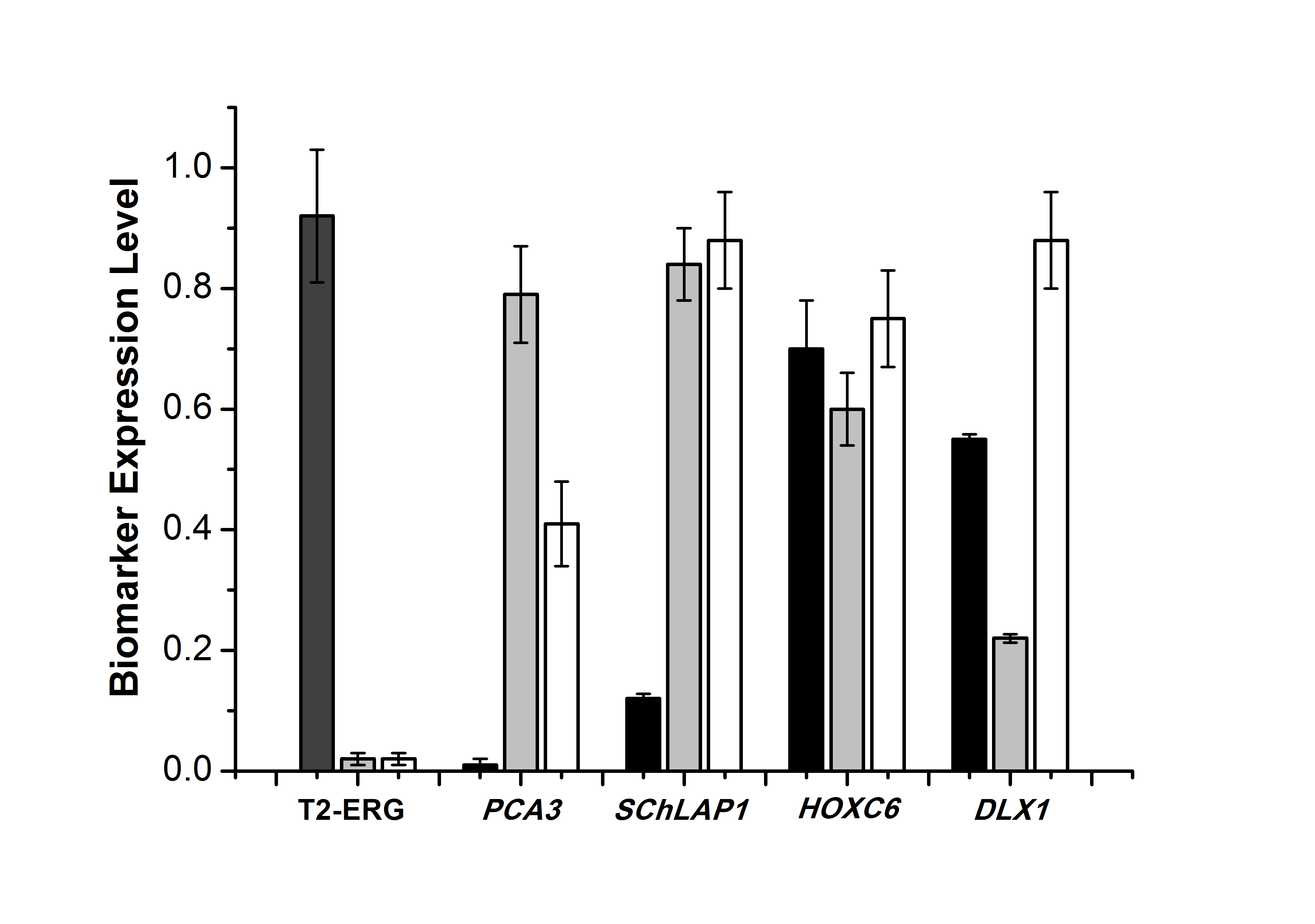


**Supplementary Figure 4.** **Validation of biomarker expressions levels in the three well-characterized prostate cancer cell lines (DuCap, LnCap, 22Rv1).** qPCR data validated the specific quantification capability of the AVATAR device.


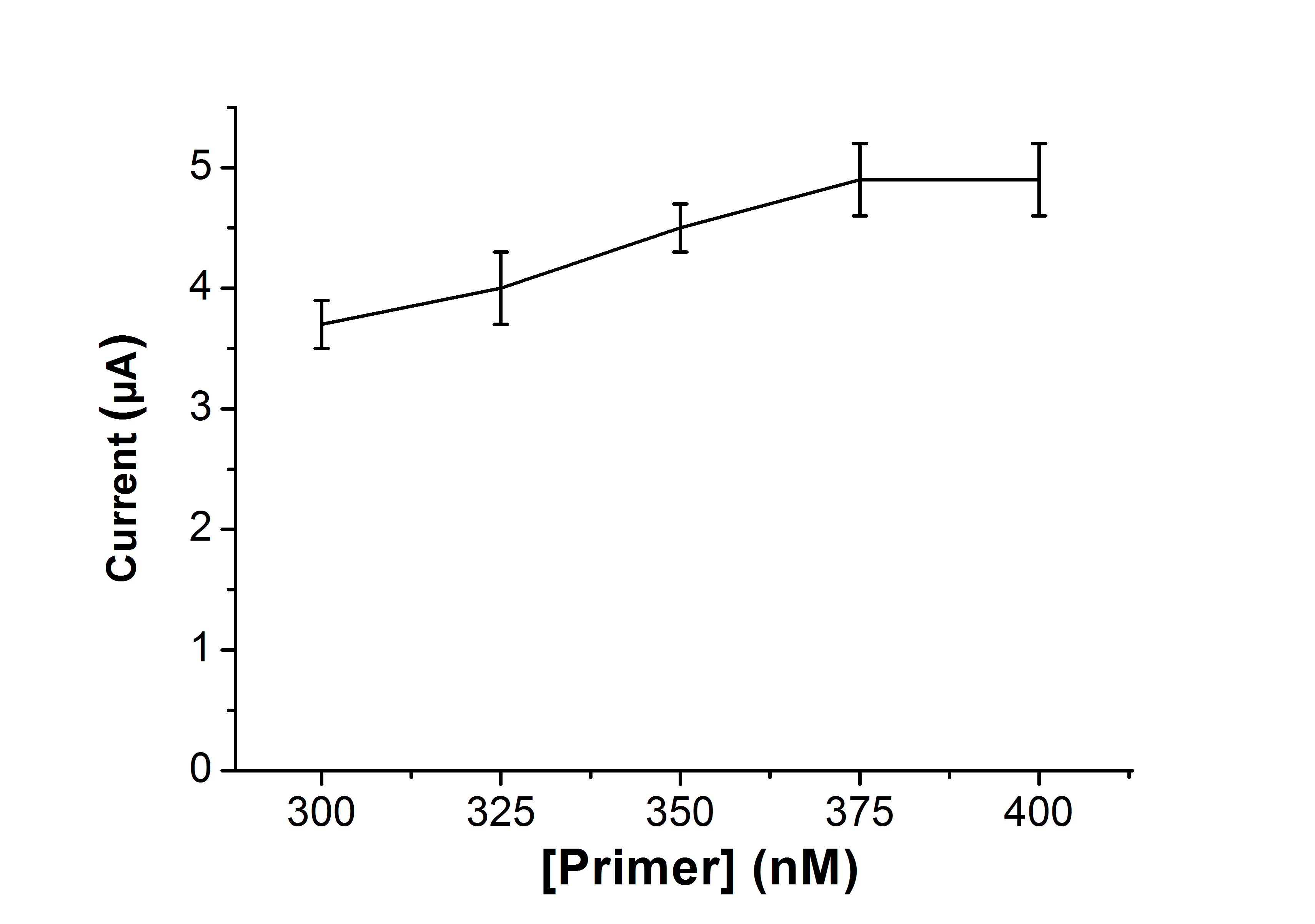


**Supplementary Figure 5. Optimization of primer concentrations (300, 325, 350, 375, 400 nM).** A 375 nM primer concentration provided the maximum current signal for biomarker detection on AVATAR.


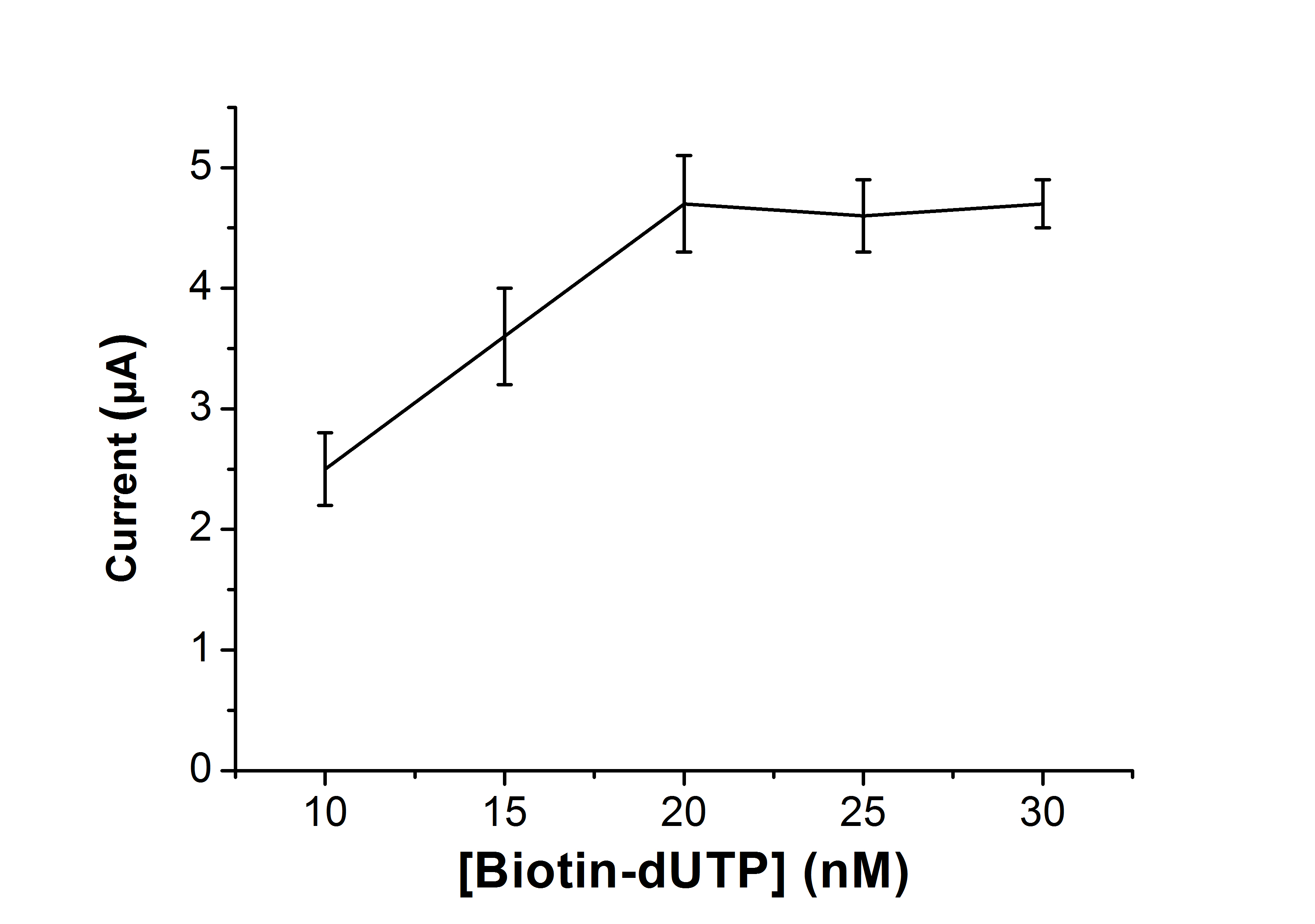


**Supplementary Figure 6. Optimization of biotin-modified uracil base concentrations (10, 15, 20, 25, 30 nM).** A 20 nM biotin-modified uracil base concentration provided the maximum current signal for biomarker detection on AVATAR.


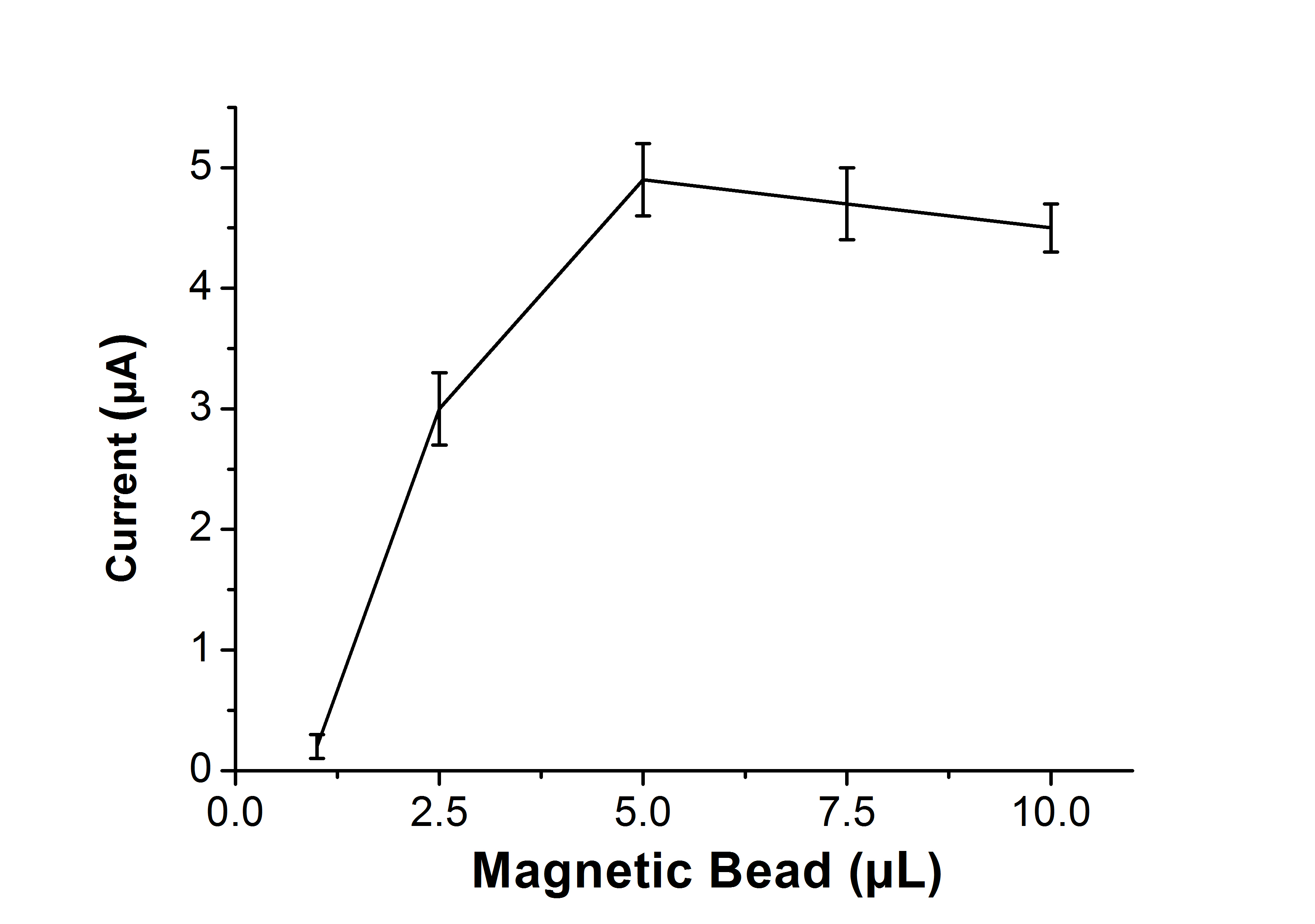


**Supplementary Figure 7. Optimization of magnetic bead volumes (1.2, 2.5, 5.0, 7.5, 10.0 µL).** A 5.0 µL magnetic bead volume provided the maximum current signal for biomarker detection on AVATAR.


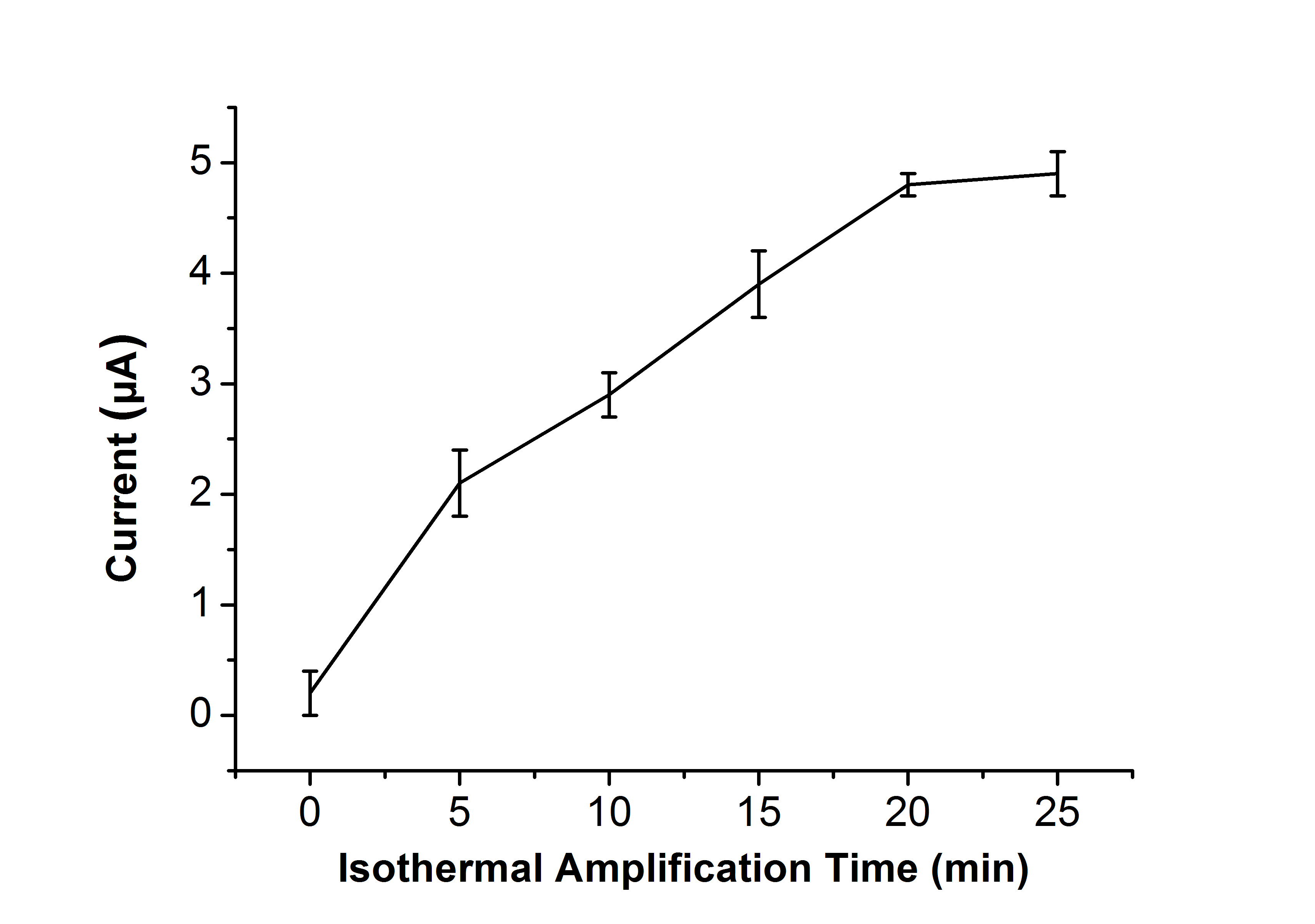


**Supplementary Figure 8.** **Optimization of isothermal amplification times (0, 5, 10, 15, 20, 25 min).** A 20 min isothermal amplification time provided the maximum current signal for biomarker detection on AVATAR.


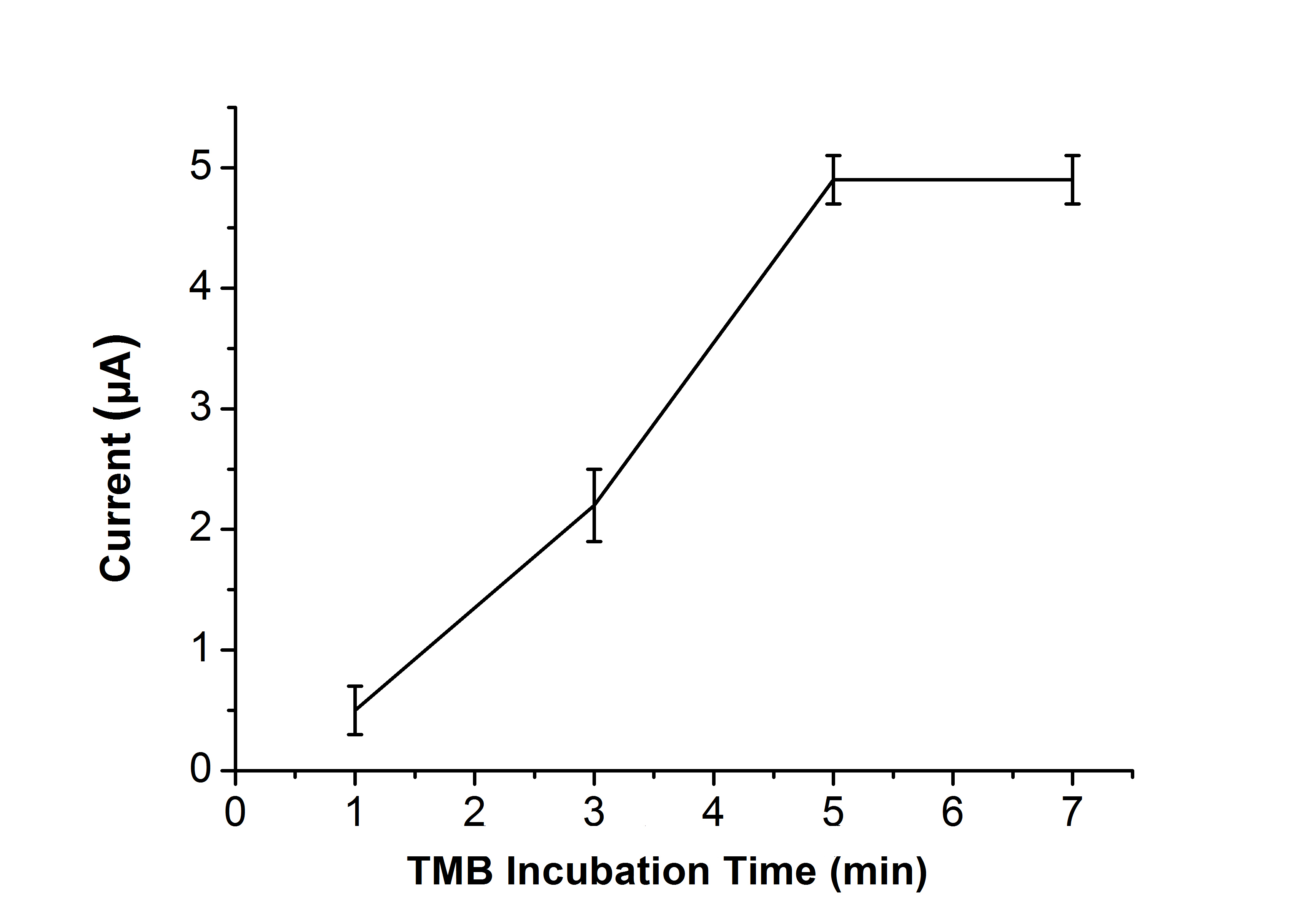


**Supplementary Figure 9.** **Optimization of TMB (3,3′,5,5′-tetramethylbenzidine) incubation times** **(1, 3, 5, 7 min).** A 5 min TMB incubation time provided the maximum current signal for biomarker detection on AVATAR.


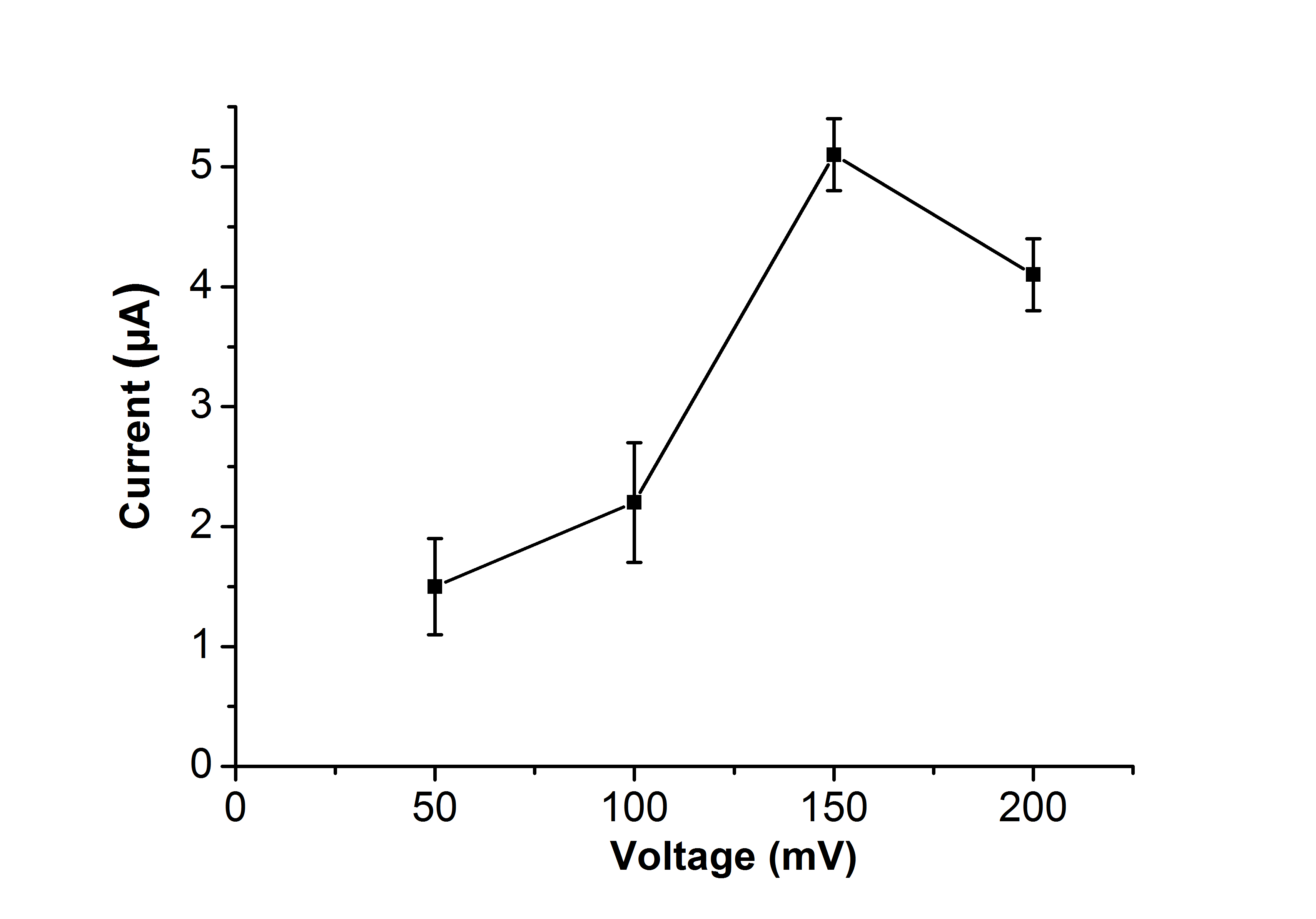
 **Supplementary Figure 10.** **Optimization of voltages for chronoamperometric readout (50, 100, 150, 200 mV).** A constant voltage of 150 mV provided the maximum current signal for biomarker detection on AVATAR.


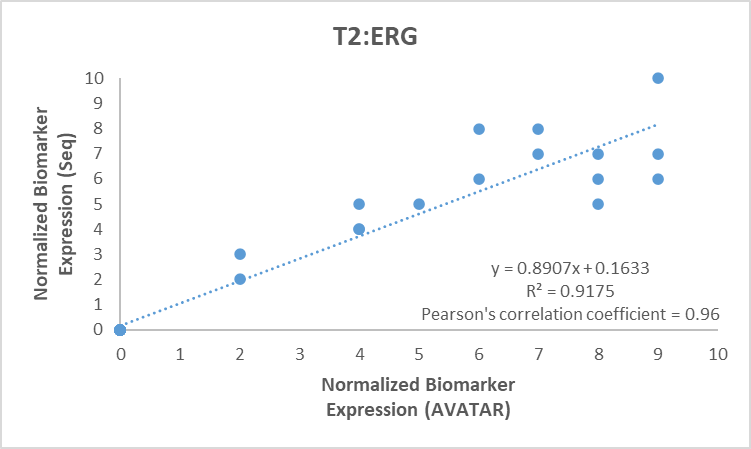

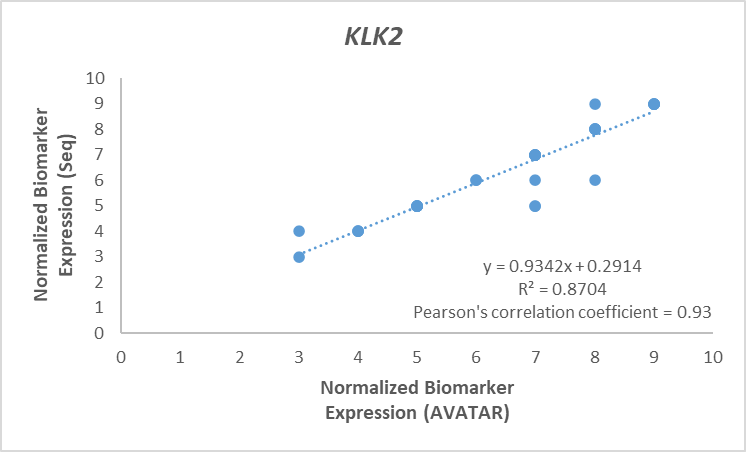


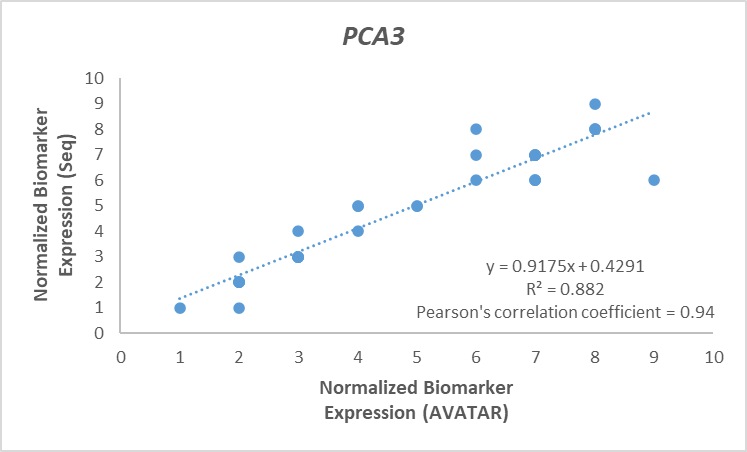


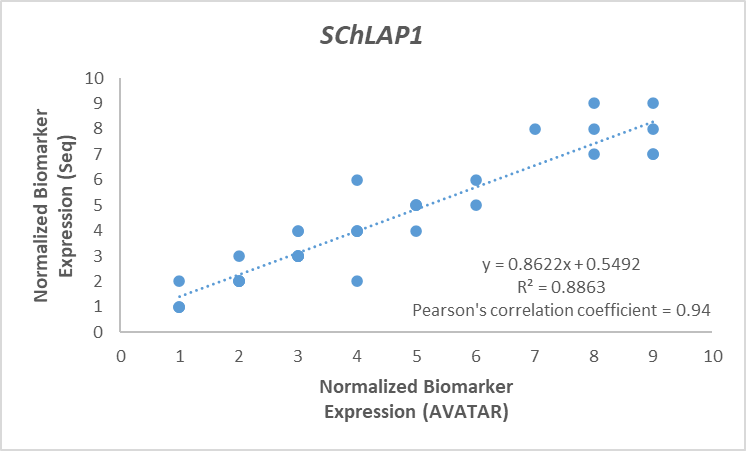


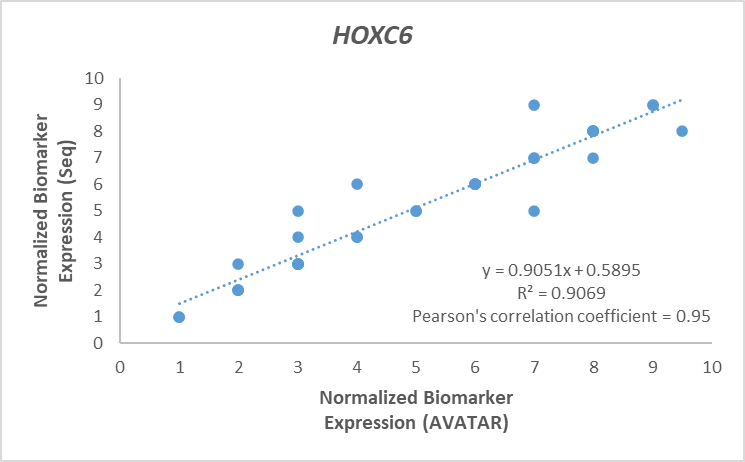


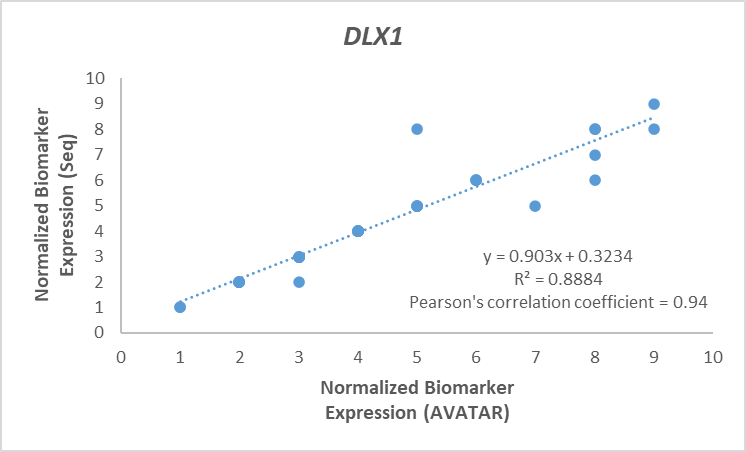


**Supplementary Figure 11. Scatter plots per gene between AVATAR and targeted RNA-sequencing measurements in clinical tissue specimens.** The linear association, slope and Pearson’s correlation coefficient values of measurement correlation (*n* =35).


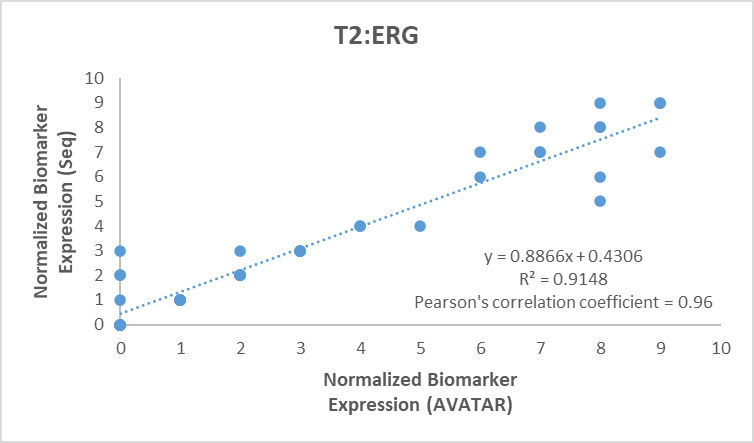

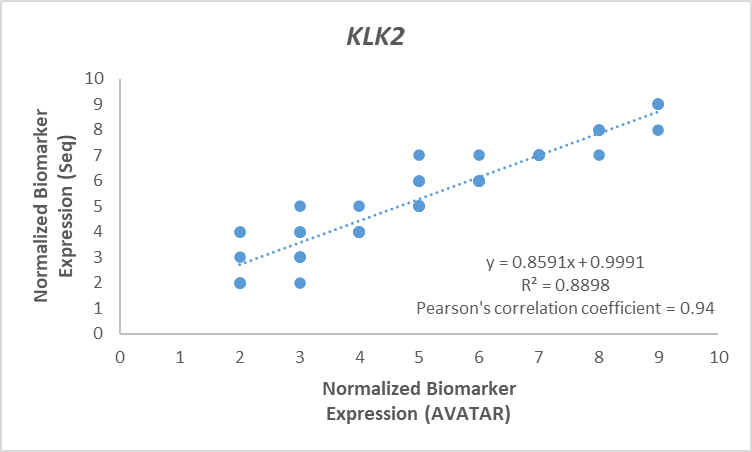


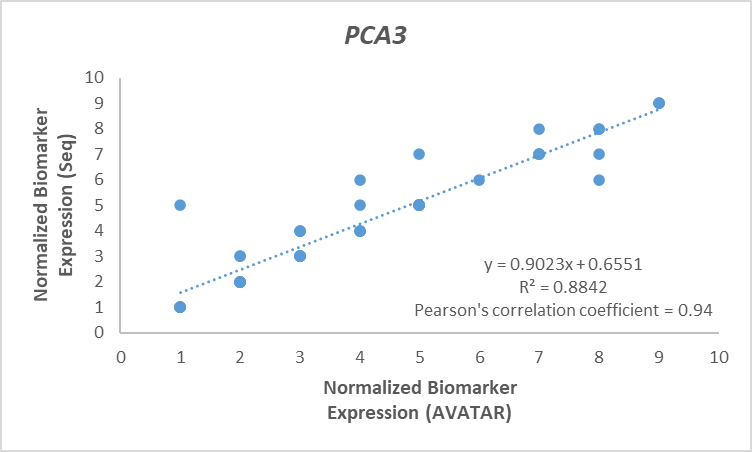


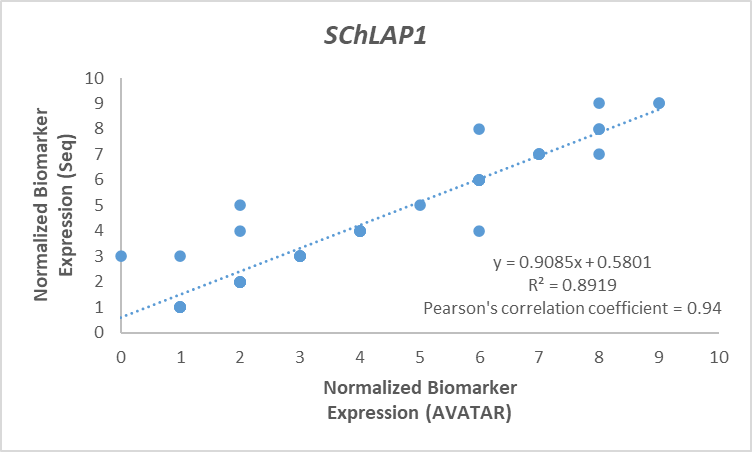


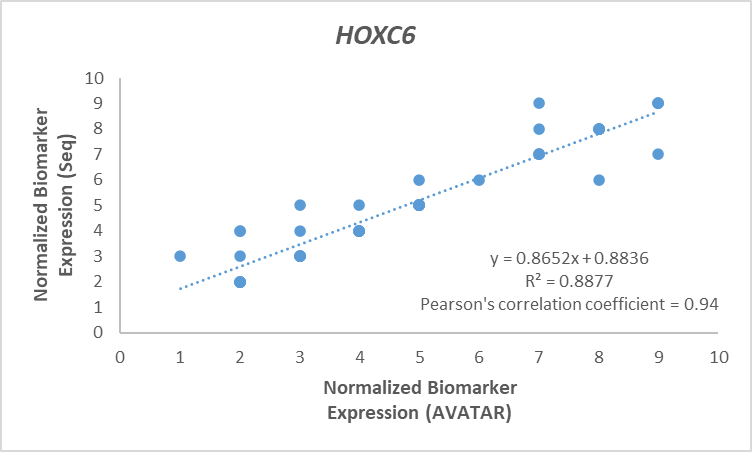


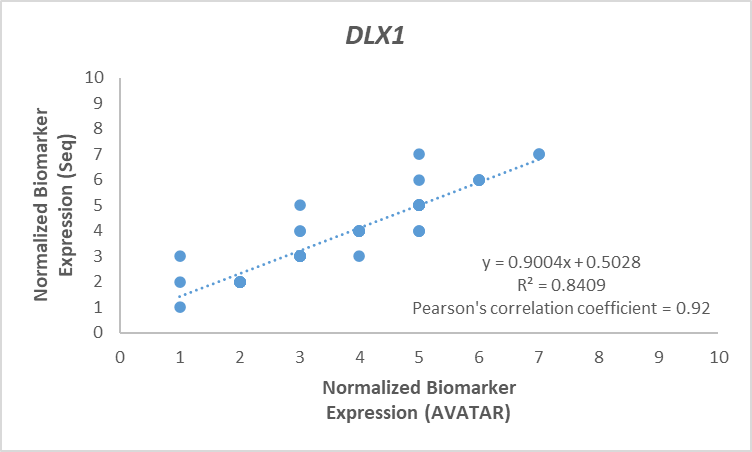


**Supplementary Figure 12. Scatter plots per gene between AVATAR and targeted RNA-sequencing measurements in clinical urinary specimens.** The linear association, slope and Pearson’s correlation coefficient values of measurement correlation (*n* = 48).


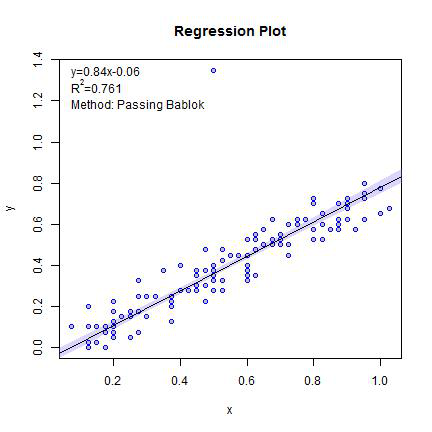

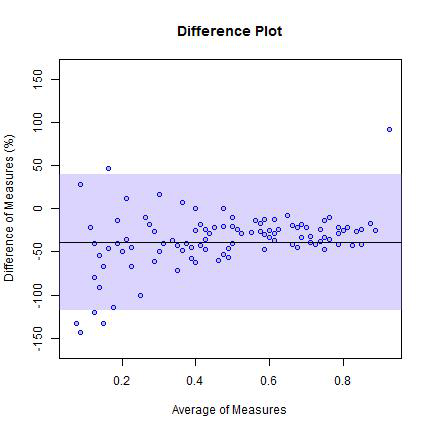


**Supplementary Figure 13.** **Passing−Boblok regression analysis of the measurements from AVATAR versus qRT-PCR in the training cohort.** Regression line equation: y = 0.84 x – 0.06 (0.84 for slope and -0.06 for intercept), *R^2^* = 0.761 (relationship between AVATAR and qRT-PCR data is linear).


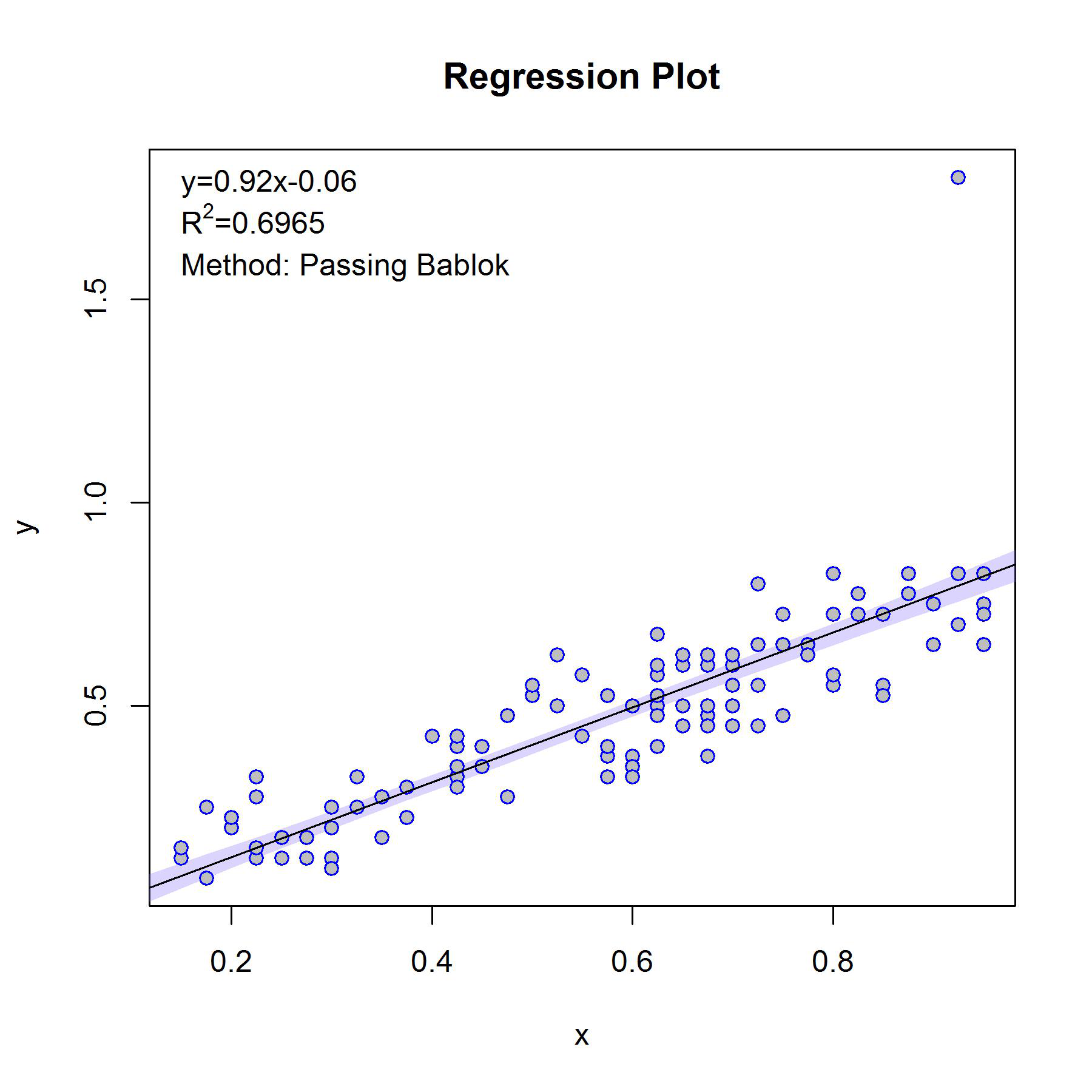

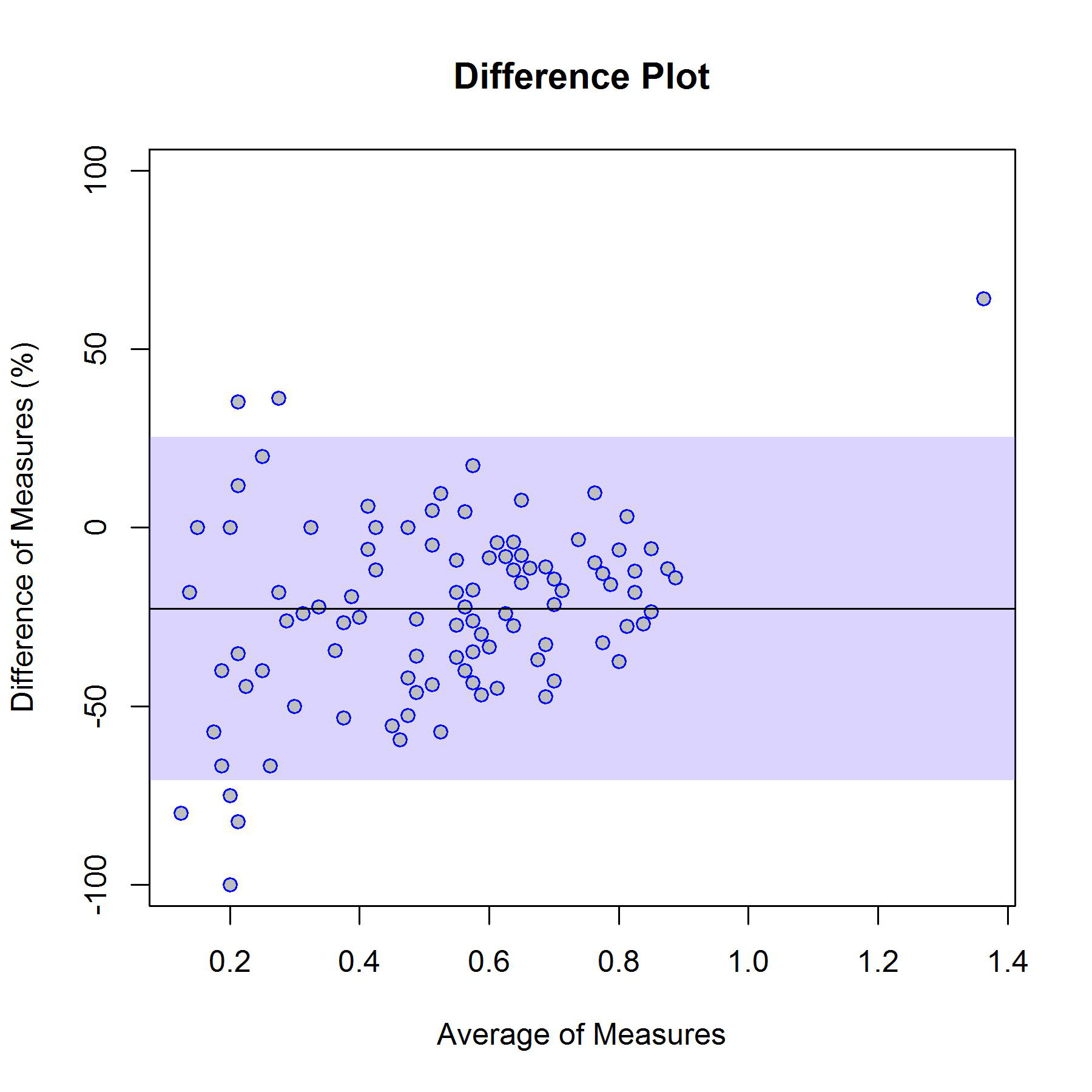


**Supplementary Figure 14.** **Passing−Boblok regression analysis of the measurements from AVATAR versus qRT-PCR in the validation cohort.** Regression line equation: y = 0.92 x – 0.06 (0.92 for slope and -0.06 for intercept), *R^2^* = 0.697 (relationship between AVATAR and qRT-PCR data is linear).


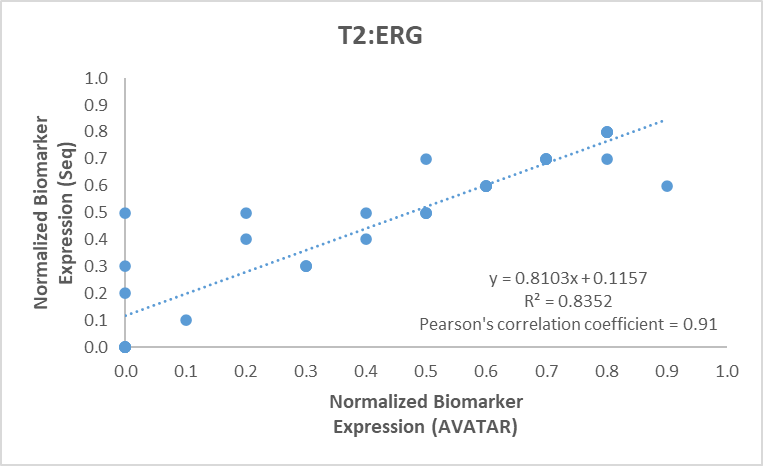

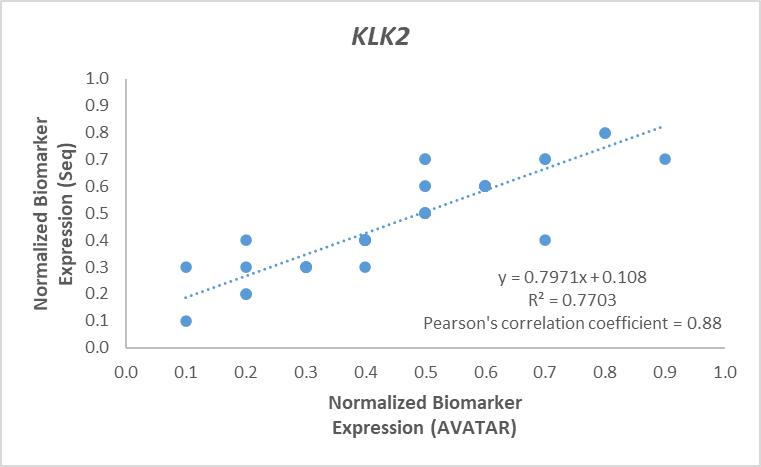


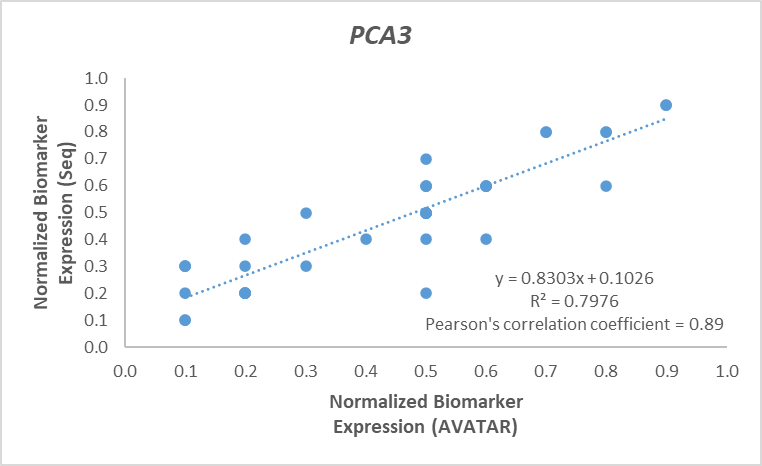


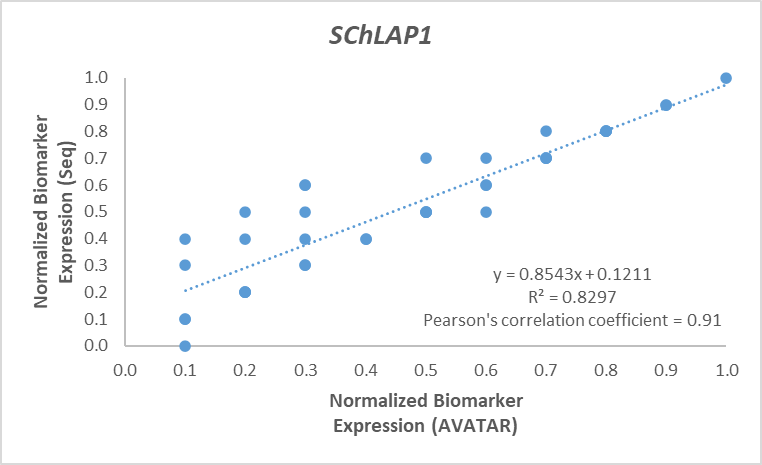


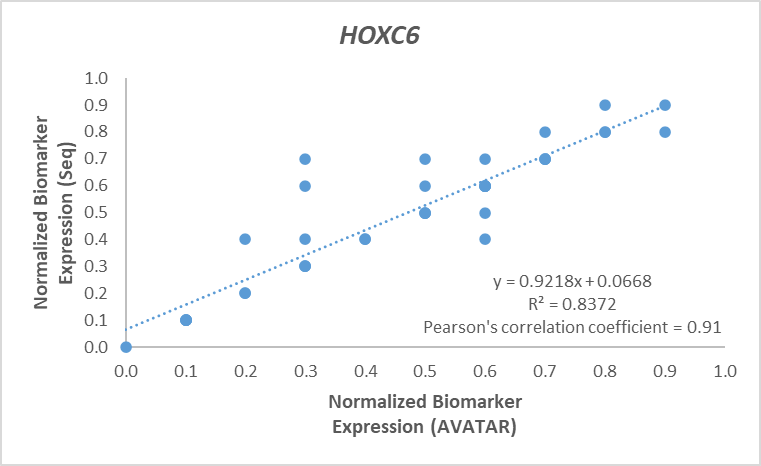


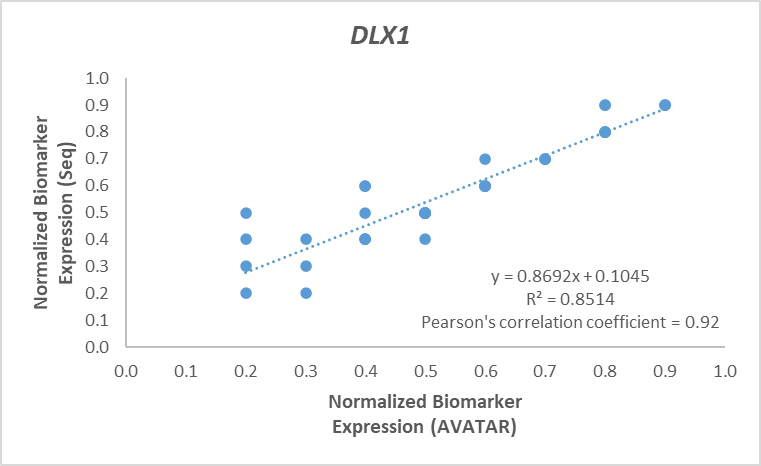


**Supplementary Figure 15. Scatter plots per gene between AVATAR and whole transcriptomic sequencing measurements in clinical urinary specimens.** The linear association, slope and Pearson’s correlation coefficient values of measurement correlation (*n* = 39).


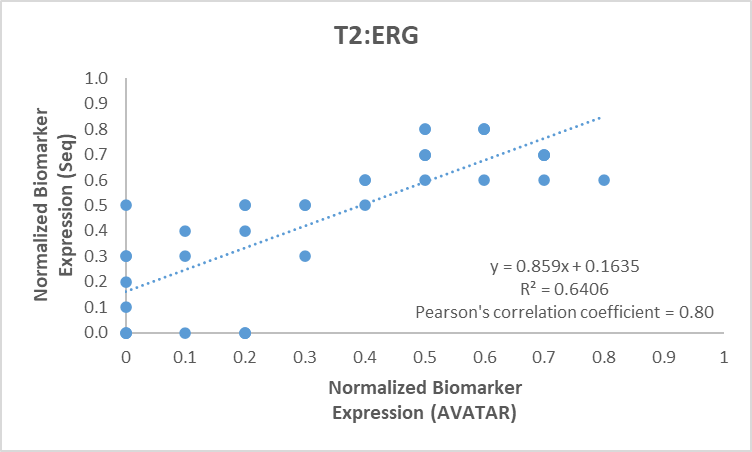

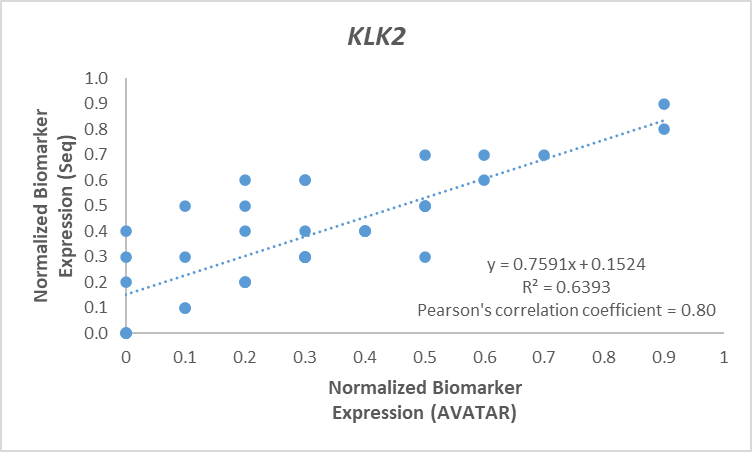


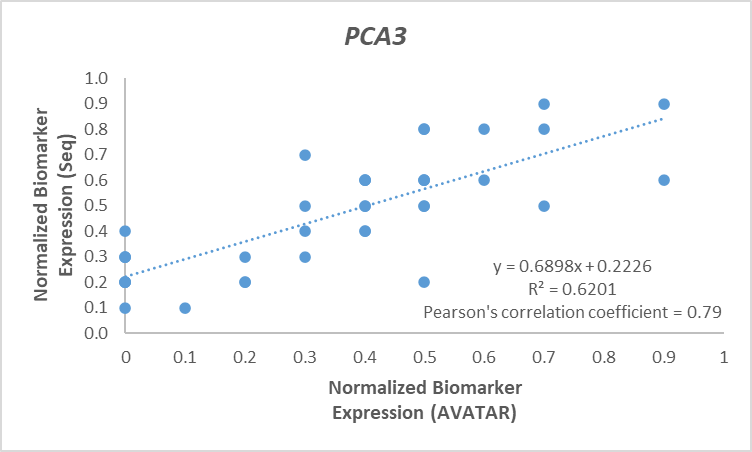


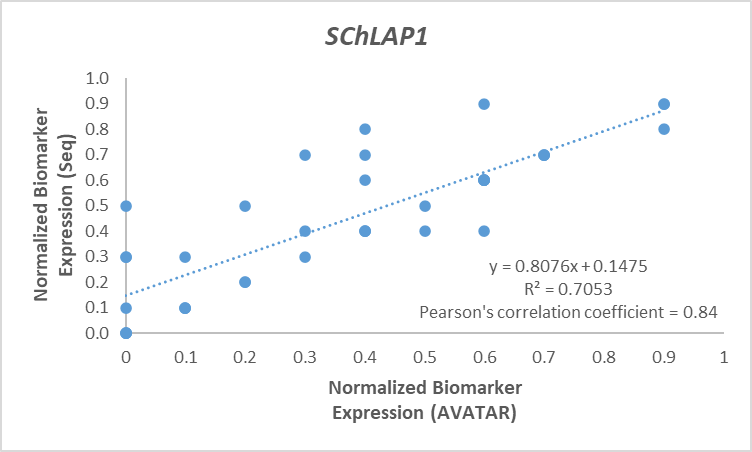


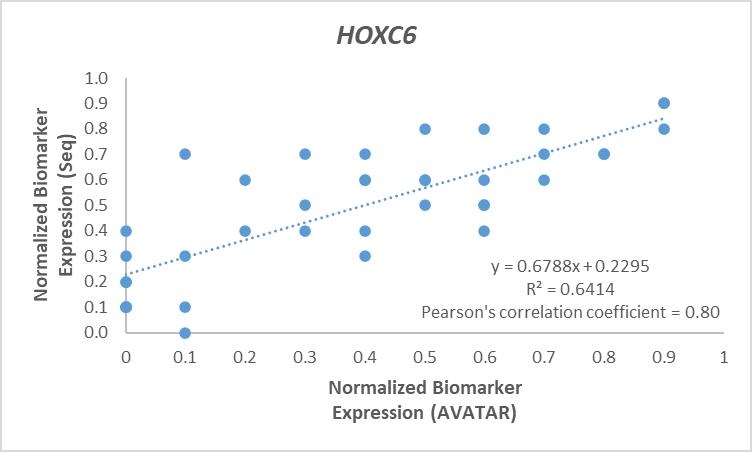


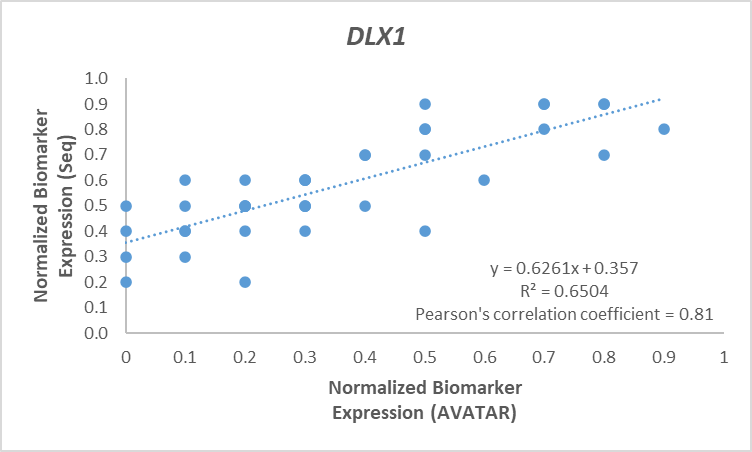


**Supplementary Figure 16. Scatter plots per gene between AVATAR and whole transcriptomic sequencing measurements in clinical plasma specimens.** The linear association, slope and Pearson’s correlation coefficient values of measurement correlation (*n* = 39).

**Supplementary Table 1.** Primer sequences used in experiments.

| **Oligos** | **5'-Sequence-3'** |
| --- | --- |
| T2:ERG Forward Primer | ATTTAGGTACAACTCTTTCCCTCGTC |
| T2:ERG Reverse Primer | TGTATAGGAATCCCACTGAATTTTTC |
| *PCA3* Forward Primer | CCTGATGATACAGAGGTGAG |
| *PCA3* Reverse Primer | GCACAGGGCGAGGCTCATCG |
| *SChLAP1* Forward Primer | GAGACCAAGAACCCACCAGTTCTGGACACA |
| *SChLAP1* Reverse Primer | CATCTGGCACTTCTTCCCCAGTCATTCCAT |
| *HOXC6* Forward Primer | CTCTCTCCCAGGCTCTTCCT |
| *HOXC6* Reverse Primer | TTGGGGAGGACGTCCGAG |
| *DLX1* Forward Primer | TGGAGGACCCAGGTCAAGAT |
| *DLX1* Reverse Primer | TAGCTTCTTGGTGCGCTGAA |
| *KLK2* Forward Primer | GGGGGTCCACTTGTCTGTAA |
| *KLK2* Reverse Primer | GGTGAGTTCCAAGCTTCAGG |

**Supplementary Table 2.** Comparisons of AVATAR with recently reported isothermal electrical signaling-based assays for circulating RNA detection in clinical cancer specimens.

| **Isothermal Electrochemical Assays** | **Target Type** | **Sample Matrix** | **Detection Limit** | **Dynamic Range** | **Assay Time** | **Portability** |
| --- | --- | --- | --- | --- | --- | --- |
| AVATAR | Prostate cancer mRNA & lncRNA | Urine, Plasma, Tissue | 100 copies | 10^2^-10^5^ copies | 55 min | Screen-printed electrodes & palm-sized potentiostat |
| Alternating current electrohydrodynamic nanomixing chip [Ref. 42] | Prostate cancer mRNA, lncRNA & miRNA | Urine | 10^4^ copies | 10^4^-10^8^ copies | 60 min | Microelectrodes & lab-based potentiostat |
| Electrochemical clamp assay chip [Ref. 43] | Lung cancer and melanoma mRNA | Serum | 1 fg (10 copies)/μL | 1 fg/μL -100 pg/μL | 35 min | Nanostructured microelectrodes & lab-based potentiostat |
| Locker probes & magneto-bioelectrocatalytic cycling assay [Ref. 44] | Prostate cancer mRNA | Urine, Plasma | 10 copies | 10-10^5^ copies | 70 min | Screen-printed electrodes & lab-based potentiostat |
| Ion-sensitive field-effect transistor (ISFET)-based system [Ref. 45] | Prostate cancer mRNA | Plasma | 30 copies | 10-10^7^ copies | >60 min | Handheld biosensor |

**Supplementary Table 3.** Training cohort (*n* = 124). Tumor Gleason scores for patient biopsy samples and AVATAR biomarker expression levels.

|  | **Pathology** | **T2:ERG** | ***PCA3*** | ***SChLAP1*** | ***HOXC6*** | ***DLX1*** |
| --- | --- | --- | --- | --- | --- | --- |
| **P1** | 2/18 3+4 to 60% PNI | 0.6 | 0.7 | 0.2 | 0.6 | 0.4 |
| **P2** | 9/19 4+3 | 0.8 | 0.6 | 0.7 | 0.8 | 0.7 |
| **P3** | 5/32 5+4 to 25% | 0.3 | 0.8 | 0 | 0.6 | 0.4 |
| **P4** | 3/12 3+4 to 100% | 0 | 0.5 | 0 | 0.6 | 0.3 |
| **P5** | benign | 0.2 | 0.4 | 0.1 | 0.4 | 0.1 |
| **P6** | 9/32 3+4 to 7% (3+3 to 30%) | 0.5 | 0.7 | 0.7 | 0.7 | 0.6 |
| **P7** | 7/9 4+3 | 0 | 0.4 | 0.3 | 0.5 | 0 |
| **P8** | 4/10 3+4 to 100% | 0.3 | 0.8 | 0.4 | 0.7 | 0.5 |
| **P9** | 8/14 3+4 to 90% | 0.8 | 0.8 | 0.7 | 0.9 | 0.6 |
| **P10** | 7/9 4+3 | 0.5 | 0.7 | 0.6 | 0.8 | 0.4 |
| **P11** | 4/24 3+4 to 35% | 0.7 | 0.6 | 0.8 | 0.7 | 0.8 |
| **P12** | 7/9 4+3 | 0.5 | 0.6 | 0.2 | 0.4 | 0.1 |
| **P13** | 5/16 3+4 to 70% | 0.7 | 0.8 | 0.9 | 0.7 | 0.7 |
| **P14** | 4/12 4+4 to 40% | 0 | 0.6 | 0.2 | 0.8 | 0.4 |
| **P15** | benign | 0 | 0.3 | 0.2 | 0.3 | 0 |
| **P16** | benign | 0.7 | 0.8 | 0.5 | 0.9 | 0.7 |
| **P17** | 4/10 4+3 to 80% | 0.5 | 0.5 | 0.3 | 0.5 | 0.2 |
| **P18** | 4/17 3+4 to 40% | 0.6 | 0.6 | 0.3 | 0.7 | 0.3 |
| **P19** | 3/14 3+4 to 40% | 0 | 0.3 | 0.1 | 0.2 | 0 |
| **P20** | 3/14 3+4 in 5-50% | 0.4 | 0.7 | 0.4 | 0.6 | 0.3 |
| **P21** | 3/13 3+3 in 10-40% | 0.6 | 0.5 | 0.2 | 0.6 | 0.2 |
| **P22** | 1/13 3+3 in 5% | 0.9 | 0.8 | 0.7 | 0.9 | 0.8 |
| **P23** | 5/5 max 4+4 in 10-90% | 0 | 0.4 | 0 | 0.1 | 0 |
| **P24** | benign 18 cores | 0.8 | 0.8 | 0.5 | 0.9 | 0.6 |
| **P25** | 10/19 4+4 5-30% | 0.4 | 0.6 | 0.2 | 0.5 | 0.3 |
| **P26** | 1/17 3+3 | 0.5 | 0.7 | 0.4 | 0.7 | 0.2 |
| **P27** | 4/16 3+4 | 0.9 | 0.8 | 0.6 | 0.8 | 0.6 |
| **P28** | 10/16 4+5 | 0 | 0.6 | 0.2 | 0.7 | 0.5 |
| **P29** | 2/18 atypical | 0.7 | 0.7 | 0.4 | 0.6 | 0.3 |
| **P30** | 1/18 3+3 | 0.3 | 0.5 | 0.2 | 0.6 | 0.3 |
| **P31** | 5/16 3+4 | 0 | 0.4 | 0.1 | 0.3 | 0.1 |
| **P32** | 5/16 3+4 | 0 | 0.3 | 0.2 | 0.4 | 0.2 |
| **P33** | 9/16 3+4 | 0.4 | 0.5 | 0.2 | 0.6 | 0.3 |
| **P34** | 2/16 3+3 | 0.7 | 0.7 | 0.5 | 0.8 | 0.6 |
| **P35** | 1/16 3+3 | 0 | 0.2 | 0.2 | 0.1 | 0.2 |
| **P36** | beiign 0/16 | 0.9 | 0.8 | 0.7 | 0.6 | 0.6 |
| **P37** | 4/16 4+5 | 0.3 | 0.5 | 0.3 | 0.5 | 0.4 |
| **P38** | 2/16 3+3 | 0 | 0.4 | 0.8 | 0.3 | 0.1 |
| **P39** | 4/14 3+4 | 0.6 | 0.5 | 0.2 | 0.6 | 0.1 |
| **P40** | 10/16 3+4 | 0.7 | 0.8 | 0.4 | 0.6 | 0.3 |
| **P41** | 1/14 3+3 to 8% | 0 | 0.1 | 0 | 0.1 | 0.1 |
| **P42** | all 20 cores benign | 0.8 | 0.6 | 0.5 | 0.7 | 0.6 |
| **P43** | 8/20 3+4 | 0.5 | 0.5 | 0.2 | 0.4 | 0.3 |
| **P44** | 2/18 PIN | 0.6 | 0.7 | 0.4 | 0.6 | 0.3 |
| **P45** | 5/6 4+3 | 0 | 0.2 | 0 | 0.3 | 0.1 |
| **P46** | all 17 cores benign | 0.4 | 0.4 | 0.1 | 0.5 | 0.4 |
| **P47** | 7/9 4+3 | 0.6 | 0.4 | 0.2 | 0.8 | 0.3 |
| **P48** | 7/19 3+4 | 0.6 | 0.5 | 0.2 | 0.7 | 0.4 |
| **P49** | 7/20 3+4 | 0.4 | 0.6 | 0.3 | 0.5 | 0.3 |
| **P50** | 1/16 3+3 | 0 | 0.6 | 0.2 | 0.4 | 0.3 |
| **P51** | 1/24 3+3 | 0.5 | 0.5 | 0.1 | 0.4 | 0.3 |
| **P52** | 1/15 3+3 | 0.7 | 0.4 | 0.4 | 0.4 | 0.3 |
| **P53** | 6/16 3+3 | 0 | 0.6 | 0.3 | 0.7 | 0.5 |
| **P54** | 1/18 PIN | 0.6 | 0.5 | 0.4 | 0.7 | 0.5 |
| **P55** | 5/16 3+3 | 0 | 0.2 | 0.1 | 0.1 | 0.1 |
| **P56** | all 8 benign | 0.3 | 0.4 | 0.2 | 0.3 | 0.3 |
| **P57** | benign 16 cores | 0.9 | 0.8 | 0.4 | 0.8 | 0.6 |
| **P58** | 5/5 4+3 | 0.5 | 0.7 | 0.6 | 0.7 | 0.7 |
| **P59** | 2/6 3+3 | 0.8 | 0.8 | 0.6 | 0.9 | 0.9 |
| **P60** | 9/15 4+5 | 0 | 0.2 | 0.2 | 0.4 | 0.3 |
| **P61** | all 10 cores benign | 0.4 | 0.6 | 0.4 | 0.5 | 0.3 |
| **P62** | 4/22 3+3 | 0 | 0.3 | 0.1 | 0.2 | 0.1 |
| **P63** | benign 20 cores | 0.6 | 0.6 | 0.4 | 0.7 | 0.3 |
| **P64** | 3/16 3+3 | 0.1 | 0.5 | 0.4 | 0.6 | 0.3 |
| **P65** | benign 16 cores | 0.7 | 0.8 | 0.7 | 0.6 | 0.8 |
| **P66** | 7/15 4+3 | 0 | 0.2 | 0 | 0.2 | 0.1 |
| **P67** | all 20 benign | 0.9 | 0.8 | 0.8 | 0.7 | 0.6 |
| **P68** | 3/14 4+5 | 0.4 | 0.6 | 0.6 | 0.6 | 0.7 |
| **P69** | atypical in 1/22 | 0.6 | 0.7 | 0.6 | 0.7 | 0.7 |
| **P70** | in 1/18 HG PIN | 0 | 0.5 | 0.4 | 0.7 | 0.5 |
| **P71** | 1/16 3+3 | 0 | 0.2 | 0 | 0.2 | 0.1 |
| **P72** | all 21 cores benign | 0.5 | 0.5 | 0.2 | 0.6 | 0.3 |
| **P73** | 5/21 3+4 | 0 | 0.3 | 0.2 | 0.2 | 0.1 |
| **P74** | 4/16 3+4 | 0 | 0.2 | 0.1 | 0.4 | 0.2 |
| **P75** | all 18 cores benign | 0.7 | 0.8 | 0.9 | 0.7 | 0.7 |
| **P76** | 4/11 4+5 | 0 | 0.3 | 0.1 | 0.3 | 0.1 |
| **P77** | all 21 cores benign | 0.3 | 0.2 | 0 | 0.4 | 0.2 |
| **P78** | all 18 cores benign | 0.5 | 0.6 | 0.2 | 0.3 | 0.3 |
| **P79** | all 12 cores benign | 0 | 0.3 | 0.1 | 0.2 | 0.2 |
| **P80** | all 14 cores benign | 0.7 | 0.8 | 0.7 | 0.7 | 0.6 |
| **P81** | 10/17 4+5 | 0 | 0.8 | 0.9 | 0.9 | 0.6 |
| **P82** | 4/22 5+4 | 0.7 | 0.8 | 0.1 | 0.7 | 0.7 |
| **P83** | 1/22 4+3 | 0.2 | 0.5 | 0.1 | 0.3 | 0.4 |
| **P84** | 1/12 HG PIN | 0.8 | 0.9 | 0.8 | 0.8 | 0.7 |
| **P85** | 7/12 4+5 | 0 | 0.2 | 0.1 | 0.3 | 0.2 |
| **P86** | all 24 cores benign | 0 | 0.2 | 0.1 | 0.3 | 0.2 |
| **P87** | all 26 cores benign | 0.5 | 0.5 | 0.5 | 0.7 | 0.7 |
| **P88** | 8/19 4+3 | 0.5 | 0.6 | 0.2 | 0.5 | 0.5 |
| **P89** | 7/14 3+4 | 0 | 0.4 | 0.1 | 0.6 | 0.5 |
| **P90** | 5/13 3+4 | 0 | 0.3 | 0 | 0.2 | 0 |
| **P91** | all 24 benign | 0.6 | 0.5 | 0.2 | 0.5 | 0.1 |
| **P92** | 4/12 3+4 | 0.5 | 0.6 | 0.2 | 0.5 | 0.2 |
| **P93** | 2/22 3+3 | 0.8 | 0.9 | 0.7 | 0.6 | 0.5 |
| **P94** | 6/12 4+5 | 0 | 0.4 | 0.5 | 0.4 | 0.5 |
| **P95** | 2/22 3+3 | 0.4 | 0.4 | 0.3 | 0.6 | 0.4 |
| **P96** | 2/22 3+3 | 0.7 | 0.7 | 0.2 | 0.6 | 0.2 |
| **P97** | 8/20 3+3 | 0 | 0.2 | 0.1 | 0.4 | 0.1 |
| **P98** | all 23 cores benign | 0.6 | 0.4 | 0.5 | 0.5 | 0.4 |
| **P99** | 7/18 3+4 | 0.5 | 0.4 | 0.4 | 0.6 | 0.5 |
| **P100** | 4/17 3+4 | 0.7 | 0.3 | 0.6 | 0.4 | 0.5 |
| **P101** | 3/20 3+4 | 0.8 | 0.7 | 0.5 | 0.5 | 0.6 |
| **P102** | 1/20 4+3 | 0 | 0.2 | 0.1 | 0.3 | 0.1 |
| **P103** | all 14 cores benign | 0.5 | 0.6 | 0.1 | 0.4 | 0.3 |
| **P104** | 1/19 3+3 | 0 | 0.6 | 0.2 | 0.5 | 0.2 |
| **P105** | 1/18 3+3 | 0 | 0.3 | 0.1 | 0.2 | 0.2 |
| **P106** | all 15 cores benign | 0.6 | 0.6 | 0.4 | 0.7 | 0.5 |
| **P107** | 6/20 3+4 | 0.8 | 0.9 | 0.7 | 0.5 | 0.6 |
| **P108** | 9/19 4+5 | 0 | 0.3 | 0.2 | 0.3 | 0.3 |
| **P109** | all 24 cores benign | 0.2 | 0.4 | 0.2 | 0.2 | 0.3 |
| **P110** | all 15 cores benign | 0 | 0.3 | 0.1 | 0.2 | 0.4 |
| **P111** | all 21 cores benign | 0 | 0.5 | 0.2 | 0.4 | 0.1 |
| **P112** | all 16 cores benign | 0.5 | 0.4 | 0.5 | 0.6 | 0.4 |
| **P113** | 1/16 3+3 | 0.6 | 0.5 | 0.4 | 0.6 | 0.3 |
| **P114** | 7 (8)/23 specimens (25 cores) 3+3 | 0.7 | 0.7 | 0.7 | 0.7 | 0.5 |
| **P115** | 7/18 4+3 | 0.6 | 0.8 | 0.6 | 0.8 | 0.4 |
| **P116** | 5/15 4+3 | 0.8 | 0.7 | 0.6 | 0.8 | 0.5 |
| **P117** | 4 (4)/24 specimens (25 cores) 4+3 | 0 | 0.2 | 0.2 | 0.4 | 0.2 |
| **P118** | all 16 cores benign | 0.6 | 0.4 | 0.6 | 0.6 | 0.6 |
| **P119** | all 22 cores benign | 0 | 0.3 | 0.4 | 0.3 | 0.1 |
| **P120** | 2/16 3+3 | 0 | 0.5 | 0.3 | 0.5 | 0.4 |
| **P121** | all 21 cores benign | 0 | 0.3 | 0 | 0.4 | 0.2 |
| **P122** | 6/14 4+4 | 0.8 | 0.7 | 0.5 | 0.6 | 0.3 |
| **P123** | all 15 cores benign | 0.2 | 0.4 | 0.1 | 0.2 | 0.1 |
| **P124** | 5/20 cores 3+4 | 0 | 0.5 | 0.2 | 0.5 | 0.4 |

**Supplementary Table 4.** Validation cohort (*n* = 114). Tumor Gleason scores for patient biopsy samples and AVATAR biomarker expression levels.

|  | **Pathology** | **T2:ERG** | ***PCA3*** | ***SChLAP1*** | ***HOXC6*** | ***DLX1*** |
| --- | --- | --- | --- | --- | --- | --- |
| **P1** | all 21 cores benign | 0.5 | 0.5 | 0.5 | 0.6 | 0.4 |
| **P2** | all 16 cores benign | 0.7 | 0.8 | 0.8 | 0.8 | 0.7 |
| **P3** | 1/16 3+3 | 0.7 | 0.5 | 0.6 | 0.7 | 0.5 |
| **P4** | 7 (8)/23 specimens (25 cores) 3+3 | 0 | 0.4 | 0.2 | 0.5 | 0.8 |
| **P5** | 7/18 4+3 | 0.4 | 0.6 | 0.6 | 0.7 | 0.8 |
| **P6** | 5/15 4+3 | 0.8 | 0.7 | 0.2 | 0.5 | 0.1 |
| **P7** | 4 (4)/24 specimens (25 cores) 4+3 | 0 | 0.4 | 0.1 | 0.4 | 0 |
| **P8** | all 16 cores benign | 0 | 0.6 | 0.2 | 0.4 | 0.3 |
| **P9** | 9/19 4+3 | 0 | 0.5 | 0.3 | 0.3 | 0.2 |
| **P10** | all 22 cores benign | 0 | 0.3 | 0.1 | 0.4 | 0.1 |
| **P11** | 2/16 3+3 | 0.5 | 0.6 | 0.5 | 0.6 | 0.4 |
| **P12** | all 21 cores benign | 0.7 | 0.7 | 0.4 | 0.5 | 0.3 |
| **P13** | 6/14 4+4 | 0.2 | 0.4 | 0.2 | 0.3 | 0.6 |
| **P14** | all 15 cores benign | 0.7 | 0.6 | 0.6 | 0.7 | 0.6 |
| **P15** | 5/20 cores 3+4 | 0.5 | 0.5 | 0.5 | 0.6 | 0.4 |
| **P16** | 2/21 3+3 | 0.6 | 0.7 | 0.7 | 0.5 | 0.4 |
| **P17** | 6/17 4+5 | 0.2 | 0.4 | 0.2 | 0.3 | 0.1 |
| **P18** | 5/16 4+3 | 0.7 | 0.4 | 0.2 | 0.4 | 0.2 |
| **P19** | 6/17 3+4 | 0.8 | 0.6 | 0.3 | 0.7 | 0.3 |
| **P20** | 5 (9)/6 specimens (15 cores) 5+4 | 0.2 | 0.3 | 0.1 | 0.3 | 0.1 |
| **P21** | 2 (3)/12 specimens (34 cores) 4+5 | 0 | 0.4 | 0 | 0.3 | 0.2 |
| **P22** | all 15 cores benign | 0.6 | 0.5 | 0.5 | 0.5 | 0.4 |
| **P23** | 2/12 3+4 | 0.5 | 0.5 | 0.4 | 0.6 | 0.4 |
| **P24** | 9/13 3+4 | 0 | 0.3 | 0.2 | 0.3 | 0.1 |
| **P25** | all 16 cores benign | 0.6 | 0.5 | 0.6 | 0.7 | 0.6 |
| **P26** | 4/12 3+3 | 0.7 | 0.7 | 0.5 | 0.8 | 0.7 |
| **P27** | 4/24 3+3 | 0.7 | 0.5 | 0.5 | 0.6 | 0.5 |
| **P28** | all 12 specimens (31 cores) benign | 0.6 | 0.5 | 0.5 | 0.5 | 0.4 |
| **P29** | 9 (16)/9 specimens (16 cores) 4+5 | 0 | 0.3 | 0.1 | 0.3 | 0.1 |
| **P30** | 1/27 3+3 | 0.9 | 0.7 | 0.7 | 0.7 | 0.7 |
| **P31** | 6/19 3+4 | 0 | 0.8 | 0.7 | 0.7 | 0.6 |
| **P32** | all 16 cores benign | 0.7 | 0.4 | 0.6 | 0.6 | 0.5 |
| **P33** | 3 (9) /9 specimens (15 cores) 3+4 | 0.8 | 0.7 | 0.8 | 0.8 | 0.5 |
| **P34** | 2 (2)/ 12 specimens (26 cores) 4+3 | 0.6 | 0.5 | 0.8 | 0.5 | 0.3 |
| **P35** | 8 specimens (17 cores) benign | 0.2 | 0.6 | 0.6 | 0.7 | 0.8 |
| **P36** | 12 specimens (33 cores) benign | 0 | 0.3 | 0.2 | 0.3 | 0 |
| **P37** | 1 (1) / 11 specimens (32 cores) 3+3 | 0.4 | 0.6 | 0.5 | 0.5 | 0.4 |
| **P38** | 5/25 3+3 | 0 | 0.7 | 0.5 | 0.5 | 0.6 |
| **P39** | 2/24 3+3 | 0.8 | 0.5 | 0.4 | 0.4 | 0.5 |
| **P40** | 4 (10) /12 specimens (21 cores) 4+3 | 0.6 | 0.6 | 0.3 | 0.5 | 0.5 |
| **P41** | 5 (19 ) / 12 specimens (41 cores) 4+3 | 0 | 0.6 | 0.4 | 0.4 | 0.3 |
| **P42** | 6 (11) / 12 specimens (40 cores) 4+3 | 0.6 | 0.7 | 0.5 | 0.6 | 0.4 |
| **P43** | 4/22 3+4 | 0.5 | 0.4 | 0.7 | 0.5 | 0.4 |
| **P44** | all 32 cores benign | 0 | 0.3 | 0.1 | 0.2 | 0.1 |
| **P45** | 6/10 4+5 | 0 | 0.2 | 0.1 | 0.3 | 0 |
| **P46** | 7/13 4+4 | 0.6 | 0.7 | 0.8 | 0.7 | 0.6 |
| **P47** | 3/17 3+3 | 0 | 0.4 | 0.2 | 0.5 | 0.1 |
| **P48** | 6/16 4+5 | 0.6 | 0.5 | 0.4 | 0.7 | 0.6 |
| **P49** | 2/24 3+3 | 0.5 | 0.4 | 0.5 | 0.5 | 0.4 |
| **P50** | 4 (9)/ 13 specimens (20 cores) 4+4 | 0 | 0.5 | 0.6 | 0.5 | 0.4 |
| **P51** | 12 specimens (44 cores) all benign | 0 | 0.5 | 0.4 | 0.4 | 0.5 |
| **P52** | 2 (2) / 12 specimens (44 cores) 3+3 | 0.7 | 0.6 | 0.7 | 0.6 | 0.6 |
| **P53** | 6 (18)/ 12 specimens (46 cores) 4+3 | 0.8 | 0.8 | 0.8 | 0.6 | 0.8 |
| **P54** | 3 (3)/ 12 specimens (52 cores) 3+3 | 0.5 | 0.6 | 0.5 | 0.7 | 0.5 |
| **P55** | 1 (1)/ 12 specimens (27 cores) 3+3 | 0.4 | 0.3 | 0.1 | 0.4 | 0 |
| **P56** | 2/10 3+4 | 0.8 | 0.6 | 0.7 | 0.6 | 0.7 |
| **P57** | 9/18 4+3 | 0.6 | 0.4 | 0 | 0.6 | 0.2 |
| **P58** | 4/16 3+4 | 0 | 0.2 | 0.1 | 0.3 | 0 |
| **P59** | all 20 cores benign | 0.8 | 0.8 | 0.6 | 0.7 | 0.6 |
| **P60** | all 25 cores benign | 0.7 | 0.7 | 0.7 | 0.7 | 0.5 |
| **P61** | 13/22 4+3 | 0 | 0.3 | 0.2 | 0.4 | 0.3 |
| **P62** | benign | 0 | 0.5 | 0.4 | 0.5 | 0.3 |
| **P63** | 9/22 4+3 | 0 | 0.2 | 0.1 | 0.2 | 0.2 |
| **P64** | 2 (2)/ 12 specimens (47 cores) 3+3 | 0.7 | 0.6 | 0.5 | 0.7 | 0.5 |
| **P65** | 3 (4)/12 specimens (61 cores) 3+3 | 0.7 | 0.5 | 0.3 | 0.6 | 0.4 |
| **P66** | 4/27 3+3 | 0.7 | 0.7 | 0.4 | 0.5 | 0.3 |
| **P67** | 4/17 4+3 | 0.6 | 0.6 | 0.2 | 0.7 | 0.6 |
| **P68** | 3/21 4+5 | 0 | 0.6 | 0.2 | 0.4 | 0.4 |
| **P69** | 5/24 4+3 | 0 | 0.2 | 0.1 | 0.3 | 0 |
| **P70** | 2/13 suspicious but not diagnostic of Ca | 0.1 | 0.2 | 0.2 | 0.4 | 0.2 |
| **P71** | 12/10 (?) 5+5 | 0.4 | 0.5 | 0.4 | 0.5 | 0.2 |
| **P72** | 9/24 3+4 | 0.8 | 0.7 | 0.6 | 0.3 | 0.3 |
| **P73** | all 32 cores benign | 0 | 0 | 0.2 | 0.3 | 0.1 |
| **P74** | 7/24 4+5 | 0 | 0.4 | 0.6 | 0.5 | 0.6 |
| **P75** | 9/18 4+5 | 0.9 | 0.7 | 0.9 | 0.7 | 0.6 |
| **P76** | all 18 cores benign | 0.7 | 0.6 | 0.7 | 0.6 | 0.5 |
| **P77** | 1/22 3+4 | 0.7 | 0.6 | 0.7 | 0.5 | 0.8 |
| **P78** | all 20 cores benign prostatic tissue | 0 | 0.5 | 0.5 | 0.4 | 0.4 |
| **P79** | 7/22 4+3 | 0.4 | 0.7 | 0.5 | 0.7 | 0.4 |
| **P80** | 1/20 3+4 | 0 | 0.3 | 0.2 | 0.5 | 0.2 |
| **P81** | 1/24 3+4 | 0.7 | 0.4 | 0.8 | 0.4 | 0.6 |
| **P82** | 10/12 4+3 | 0 | 0.3 | 0.7 | 0.5 | 0.6 |
| **P83** | 2 (2)/ 14 specimens (15 cores) 3+3 | 0.8 | 0.5 | 0.5 | 0.7 | 0.7 |
| **P84** | all 16 cores benign | 0.5 | 0.5 | 0.4 | 0.4 | 0.5 |
| **P85** | all 12 specimens (37 cores) benign | 0.4 | 0.2 | 0.1 | 0.3 | 0.1 |
| **P86** | 9/26 3+4 | 0.5 | 0.6 | 0.6 | 0.6 | 0.7 |
| **P87** | 12/21 3+4 | 0.7 | 0.8 | 0.7 | 0.8 | 0.7 |
| **P88** | all 17 cores benign | 0 | 0.5 | 0.6 | 0.6 | 0.6 |
| **P89** | 5/25 3+3 | 0.6 | 0.6 | 0.4 | 0.7 | 0.6 |
| **P90** | 9/20 4+5 | 0.8 | 0.9 | 0.8 | 0.7 | 0.6 |
| **P91** | 6 (7)/ 12 specimens (17 cores) 3+4 | 0.4 | 0.4 | 0.7 | 0.5 | 0.7 |
| **P92** | 6/12 4+3 | 0.5 | 0.6 | 0.6 | 0.6 | 0.5 |
| **P93** | 1/28 3+3 | 0 | 0.5 | 0.3 | 0.5 | 0.4 |
| **P94** | 4/19 cores 3+4 | 0.6 | 0.8 | 0.6 | 0.8 | 0.8 |
| **P95** | all 24 cores benign | 0 | 0.4 | 0.4 | 0.8 | 0.7 |
| **P96** | 11/16 cores 4+3 | 0.6 | 0.5 | 0.3 | 0.4 | 0.6 |
| **P97** | 14/16 cores 4+3 | 0 | 0.4 | 0.2 | 0.5 | 0.3 |
| **P98** | 3/13 cores 4+3 | 0 | 0.3 | 0 | 0.5 | 0.2 |
| **P99** | 1/19 cores high grade prostatic intraepithelial neoplasia | 0 | 0.4 | 0.2 | 0.5 | 0.2 |
| **P100** | all 22 cores benign | 0.3 | 0.5 | 0.3 | 0.4 | 0 |
| **P101** | 18/26 cores 3+4 | 0.6 | 0.6 | 0.5 | 0.6 | 0.6 |
| **P102** | 8/30 cores 4+5 | 0 | 0.3 | 0.1 | 0.4 | 0.4 |
| **P103** | 3 (4 cores)/ 12 (30 cores) 3+4 | 0.4 | 0.4 | 0.5 | 0.6 | 0.6 |
| **P104** | 13/30 cores 4+3 | 0.3 | 0.3 | 0.4 | 0.5 | 0.2 |
| **P105** | 7/17 cores 4+5 | 0.6 | 0.7 | 0.6 | 0.7 | 0.5 |
| **P106** | 5/21 cores 3+4 | 0.5 | 0.7 | 0.7 | 0.7 | 0.6 |
| **P107** | 3/20 cores 4+3 | 0.8 | 0.7 | 0.7 | 0.8 | 0.7 |
| **P108** | 2/18 cores high grade prostatic intraepithelial neoplasia | 0.3 | 0.3 | 0.2 | 0.5 | 0.1 |
| **P109** | 8/19 cores 4+5 | 0 | 0.4 | 0.1 | 0.4 | 0 |
| **P110** | 11 (11)/ 18 (21 cores) 3+4 | 0.7 | 0.8 | 0.5 | 0.7 | 0.8 |
| **P111** | 3/20 cores high grade prostatic intraepithelial neoplasia | 0.5 | 0.6 | 0.5 | 0.5 | 0.5 |
| **P112** | all 18 cores benign | 0.6 | 0.6 | 0.2 | 0.4 | 0.4 |
| **P113** | all 21 cores benign | 0 | 0.5 | 0.7 | 0.6 | 0.6 |
| **P114** | all 20 cores benign | 0.7 | 0.4 | 0 | 0.6 | 0.5 |
